# Fluid Management, Intra-Abdominal Hypertension and the Abdominal Compartment Syndrome: A Narrative Review

**DOI:** 10.3390/life12091390

**Published:** 2022-09-06

**Authors:** Rita Jacobs, Robert D. Wise, Ivan Myatchin, Domien Vanhonacker, Andrea Minini, Michael Mekeirele, Andrew W. Kirkpatrick, Bruno M. Pereira, Michael Sugrue, Bart De Keulenaer, Zsolt Bodnar, Stefan Acosta, Janeth Ejike, Salar Tayebi, Johan Stiens, Colin Cordemans, Niels Van Regenmortel, Paul W. G. Elbers, Xavier Monnet, Adrian Wong, Wojciech Dabrowski, Philippe G. Jorens, Jan J. De Waele, Derek J. Roberts, Edward Kimball, Annika Reintam Blaser, Manu L. N. G. Malbrain

**Affiliations:** 1 Intensive Care Department, Antwerp University Hospital, 2650 Edegem, Belgium; 2 Faculty Medicine and Pharmacy, Vrije Universiteit Brussel (VUB), 1050 Brussels, Belgium; 3 Discipline of Anesthesiology and Critical Care, School of Clinical Medicine, University of KwaZulu-Natal, Durban 4001, South Africa; 4 Adult Intensive Care, John Radcliffe Hospital, Oxford University Hospitals NHS Foundation Trust, OX3 9DU Oxford, UK; 5 Intensive Care Department, University Hospital Brussels, Universitair Ziekenhuis Brussel, Laarbeeklaan 101, 1090 Brussels, Belgium; 6 Emergency Medicine Department, Ziekenhuis Oost-Limburg, Schiepse Bos 6, 3600 Genk, Belgium; 7 Department of Anesthesiology and Intensive Care, Ospedale di Circolo e Fondazione Macchi, University of Insubria, 21100 Varese, Italy; 8 Departments of Critical Care Medicine and Surgery, The Trauma Program, University of Calgary, Victoria, BC V8W 2Y2, Canada; 9 The TeleMentored Ultrasound Supported Medical Interventions (TMUSMI) Research Group, Calgary, AB T3H 3W8, Canada; 10 Department of Surgery, Health Applied Sciences, Vassouras University, Vassouras 27700, Brazil; 11 Campinas Holy House Residency Program, Terzius Institute, Campinas 13010, Brazil; 12 Donegal Clinical Research Academy and Emergency Surgery Outcome Advancement Project (eSOAP), F94 A0W2 Donegal, Ireland; 13 Department of Intensive Care, Fiona Stanley Hospital; Professor at the School of Surgery, The University of Western Australia, Perth, WA 6907, Australia; 14 Department of Intensive Care at SJOG Murdoch Hospital, Murdoch, WA 6150, Australia; 15 Consultant General Surgeon, Letterkenny University Hospital, F92 AE81 Letterkenny, Ireland; 16 Department of Clinical Sciences, Lund University, Box 117, SE-221 00 Lund, Sweden; 17 Department of Pediatrics, Loma Linda University Children’s Hospital, Loma Linda, CA 92354, USA; 18 Faculty of Engineering, Department of Electronics and Informatics, Vrije Universiteit Brussel (VUB), 1040 Etterbeek, Belgium; 19 Department of Intensive Care, AZ Sint-Maria Hospital, 1500 Halle, Belgium; 20 Department of Intensive Care Medicine, Campus Stuivenberg, Ziekenhuis Netwerk Antwerpen, 2050 Antwerp, Belgium; 21 Department of Intensive Care Medicine, Laboratory for Critical Care Computational Intelligence, Amsterdam Medical Data Science (AMDS), Amsterdam UMC, Vrije Universiteit, 1081 Amsterdam, The Netherlands; 22 Groupe de Recherche Clinique CARMAS, Université Paris-Saclay, Service de Médecine Intensive-Réanimation, Hôpital de Bicêtre, FHU SEPSIS, 94275 Le Kremlin-Bicêtre, France; 23 Department of Critical Care, King’s College Hospital NHS Foundation Trust London, London SE5 9RS, UK; 24 First Department of Anaesthesiology and Intensive Therapy, Medical University of Lublin, Jaczewskiego 8, 20-954 Lublin, Poland; 25 University of Antwerp, Laboratory of Experimental Medicine and Pediatrics (LEMP), 2000 Antwerpen, Belgium; 26 Intensive Care Unit, University Hospital Ghent, 9000 Ghent, Belgium; 27 Division of Vascular and Endovascular Surgery, Department of Surgery, Faculty of Medicine, University of Ottawa, Ottawa, ON K1N 1H3, Canada; 28 Department of Surgery and Critical Care, U Health OND&T, Salt Lake City, UT 84105, USA; 29 Department of Surgical Critical Care SLC VA Medical Center, Salt Lake City, UT 84148, USA; 30 Department of Anesthesiology and Intensive Care, University of Tartu, 50090 Tartu, Estonia; 31 Department of Intensive Care Medicine, Lucerne Cantonal Hospital, 6110 Lucerne, Switzerland; 32 Medical Data Management, Medaman, 2440 Geel, Belgium; 33 International Fluid Academy, 3360 Lovenjoel, Belgium

**Keywords:** fluid therapy, abdominal hypertension, abdominal compartment syndrome, open abdomen, crystalloids, colloids, hypertonic, resuscitation, maintenance, sepsis

## Abstract

Background: General pathophysiological mechanisms regarding associations between fluid administration and intra-abdominal hypertension (IAH) are evident, but specific effects of type, amount, and timing of fluids are less clear. Objectives: This review aims to summarize current knowledge on associations between fluid administration and intra-abdominal pressure (IAP) and fluid management in patients at risk of intra-abdominal hypertension and abdominal compartment syndrome (ACS). Methods: We performed a structured literature search from 1950 until May 2021 to identify evidence of associations between fluid management and intra-abdominal pressure not limited to any specific study or patient population. Findings were summarized based on the following information: general concepts of fluid management, physiology of fluid movement in patients with intra-abdominal hypertension, and data on associations between fluid administration and IAH. Results: We identified three randomized controlled trials (RCTs), 38 prospective observational studies, 29 retrospective studies, 18 case reports in adults, two observational studies and 10 case reports in children, and three animal studies that addressed associations between fluid administration and IAH. Associations between fluid resuscitation and IAH were confirmed in most studies. Fluid resuscitation contributes to the development of IAH. However, patients with IAH receive more fluids to manage the effect of IAH on other organ systems, thereby causing a vicious cycle. Timing and approach to de-resuscitation are of utmost importance, but clear indicators to guide this decision-making process are lacking. In selected cases, only surgical decompression of the abdomen can stop deterioration and prevent further morbidity and mortality. Conclusions: Current evidence confirms an association between fluid resuscitation and secondary IAH, but optimal fluid management strategies for patients with IAH remain controversial.

## 1. Background

Over the past two decades, the pathophysiological consequences of intra-abdominal hypertension (IAH) and abdominal compartment syndrome (ACS) have received more research and awareness. The Abdominal Compartment Society (WSACS, www.wsacs.org (accessed on: 26 May 2022)) has set out clear definitions for intra-abdominal hypertension (subclassified into primary and secondary) and abdominal compartment syndrome, including the importance of concepts such as abdominal perfusion pressure (APP) [1,2,3,4].

Elevation of IAP leads to compromise in several organ systems, including cardiovascular (decreasing preload, increasing afterload, and decreasing cardiac output), respiratory (elevated diaphragm, decreased compliance, decreased functional residual capacity), central nervous system (intracranial hypertension due to functional obstruction of cerebral venous outflow), renal (compression of both the renal veins and arteries), and the gastrointestinal system (GIT) [5,6,7,8,9,10,11,12,13,14]. The effect on the GIT is multiple and includes mesenteric vein compression, decreased perfusion, intestinal edema, bacterial translocation, and disruption of the gut microbiome and immune system [3].

The pathophysiological impact of elevated IAP on the various organ systems mimics a state like sepsis, with loss of vasomotor tone and dysfunction of the intercellular junctions of the endothelium. Fluid resuscitation is often the first choice to restore hemodynamic stability. However, administration of large volumes of intravenous fluids may paradoxically result in ACS. The increased abdominal pressure stimulates anti-diuretic hormone (ADH) release, further promoting fluid retention [4]. Dabrowski et al., documented a significant correlation between IAP and extravascular water content in critically ill patients and patients undergoing prolonged elective surgery [5]. Reintam et al., showed that mortality among patients with secondary IAH was significantly higher than among patients with primary IAH [6], whereas a meta-analysis looking at various risk factors for IAH identified fluid balance as an independent predictor for IAH [7].

The effect of intravenous fluid administration on IAP has been studied, however, the effects of fluids on IAP in different patient populations and conditions remain largely unexplored.

The effect of pressures on neighboring anatomical compartments highlights the importance of appropriate management of patients with IAH or elevated pressures in adjacent compartments (abdomen, thorax, skull) [10,11,12,13]. In 2007, Thomas Scalea was the first to suggest the complex and constant interplay of elevated pressure between different compartments [8]. The poly-compartment syndrome (PCS) as “terminus technicus” coined by Malbrain has been well described in the medical literature [10,11,12,13]. Genuine PCS is a rare, but life-threatening condition, when two or more compartments have simultaneously elevated pressures. Releasing the pressure of one of the affected compartments usually improves the clinical scenario [15,16].

**Figure 1:**
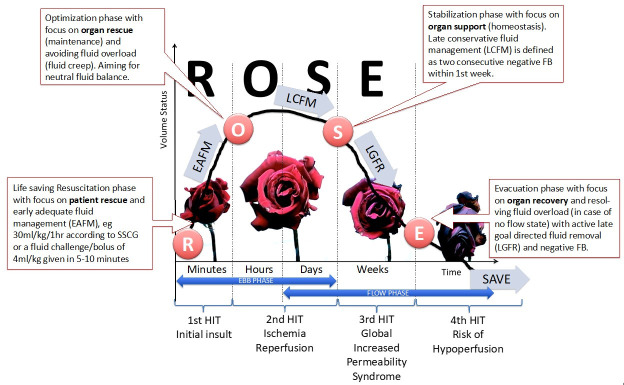
The four hits of shock. Graph showing the four-hit model of shock with evolution of patients’ cumulative fluid volume status over time during the five distinct phases of resuscitation: Resuscitation (R), Optimization (O), Stabilization (S), and Evacuation (E) (ROSE), followed by a possible risk of Hypoperfusion in case of too aggressive de-resuscitation. On admission patients are hypovolemic, followed by normovolemia after fluid resuscitation (EAFM, early adequate fluid management), and possible fluid overload, again followed by a phase going to normovolemia with late conservative fluid management (LCFM) and late goal directed fluid removal (LGFR) or de-resuscitation. In the case of hypovolemia, O_2_ cannot get into the tissue because of convective problems; in the case of hypervolemia, O_2_ cannot get into the tissue because of diffusion problems related to interstitial and pulmonary edema, gut edema (ileus and abdominal hypertension). Adapted according to the Open Access CC BY License 4.0 from Malbrain et al., with permission [17].

The goals of treatment for PCS are:

1. To reduce the pressure in the compartment by improving compliance (e.g., muscle relaxation) and, or opening different compartments (e.g., through escharotomy or decompressive surgery).
2. Individualized fluid management strategies and supportive therapy.
3. Apply the concepts of the four stages of fluid resuscitation (ROSE model, Figure 1) [17].
4. Avoid the adverse effects of ischemia-reperfusion after surgical decompression [11,12,13].

As the available data is scarce, this scoping review aims to describe the impact of fluid resuscitation on the development of intra-abdominal hypertension. Firstly, current knowledge on the pathophysiology of fluid administration is summarized, focusing on specific aspects related to increased IAP. Secondly, studies addressing fluid management in subjects with IAH are summarized and discussed.

## 2. Methods

Methods for inclusion, analysis and reporting of results were according to recommendations from the preferred reporting items for systematic reviews and meta-analyses (PRISMA).

### Search Strategy

**Figure 2:**
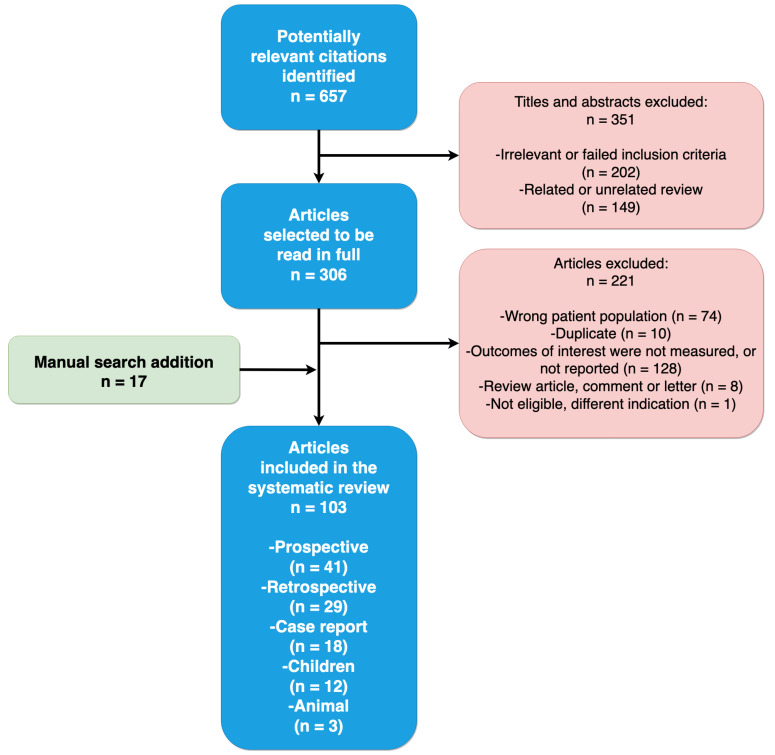
Flowchart of literature review and selection of included publications.

Two investigators performed a literature search for articles between 1950 and May 2021 using Scopus and PubMed electronic databases. We used the following search terms: (“abdominal hypertension” OR “abdominal compartment syndrome” OR “abdominal pressure”) AND (“fluid therapy” OR “fluid resuscitation” OR “risk factor”). The search was limited to English-language articles. PubMed search formula included (“intra-abdominal hypertension” [All Fields] OR “intra-abdominal pressure” [All Fields]) OR “abdominal compartment syndrome” [All Fields]) AND (“fluid” [All Fields] OR “resuscitation” [All Fields]). Identified citations had their titles and abstracts independently screened for the relationship between fluid therapy and IAH (Figure 2). We used the following inclusion criteria: (1) No age limitation and animal studies included; (2) studies that examined the association between fluid resuscitation and IAH or ACS; (3) IAH diagnosed using trans-bladder pressure measurements; and (4) no limitation to the type of study design. Disagreements between investigators regarding study inclusion were resolved by consensus. Reference lists of these papers, and related articles featured in PubMed, were screened to identify additional studies not identified through the initial literature search.

The same two authors extracted the following data independently (as tabulated in the manuscript): (1) design and setting; (2) study participant diagnosis (for example, trauma, burn, severe acute pancreatitis); (3) type and amount of fluid administered; (4) IAP measurement in mmHg; (5) patient outcomes; (6) management/intervention of IAH or ACS.

All relevant studies underwent a full-text assessment, and data were extracted into tables according to the study type/design. Studies and hypotheses on pathophysiological mechanisms are summarized as narrative text. One hundred and three of the 657 potentially relevant publications identified during the literature search were included (Figure 2).

## 3. Results

### 3.1. Data on Associations between Fluid Administration and IAH

#### 3.1.1. Study selection and characteristics

Among 764 unique citations, 103 studies enrolling 12015 critically ill adults, 107 critically ill children, and 104 animals met the inclusion criteria (Figure 2) [5,18,19,20,21,22,23,24,25,26,27,28,29,30,31,32,33,34,35,36,37,38,39,40,41,42,43,44,45,46,47,48,49,50,51,52,53,54,55,56,57,58,59,60,61,62,63,64,65,66,67,68,69,70,71,72,73,74,75,76,77,78,79,80,81,82,83,84,85,86,87,88,89,90,91,92,93,94,95,96,97,98,99,100,101,102,103,104,105,106,107,108,109,110,111,112,113,114,115,116,117,118,119]. Among the 103 included studies, 3 were randomized controlled trials (RCTs) (Table 1), 39 prospective observational studies (Table 1), 29 retrospective studies (Table 2), 17 case reports in adult patients (Table 3), two observational studies and 10 case reports in children (Table 4), and three animal studies (Table 5).

From the analyzed and discussed studies, twenty included burn patients [18,19,20,21,22,23,24,25,26,27,28,29,30,31,32,33,34,35,36,37], seven included severe acute pancreatitis (SAP) patients [40,41,42,43,44,45,46], thirty included trauma patients [38,39,47,48,49,50,51,52,53,54,55,56,57,58,59,60,61,62,63,64,65,66,67,68,69,70,71,73,74,75], fourteen included medical patients [70,72,76,77,78,79,80,81,82,83,84,85,86,87], seventeen included surgical patients [63,88,89,90,91,92,93,94,95,96,97,98,99,100,101,102,103], fourteen included medical-surgical (and critically ill) patients [5,104,105,106,107,108,109,110,111,112,113,114,115,116].

Pooled analysis was not possible because of the heterogeneity in study populations and data, and the lack of details on IAP measurement techniques.

Several above-cited studies have investigated the relationship between the volume of intravenous fluids administered and their effect on IAP. Most of these trials reported an association between the volume of intravenous fluids administered and the rise in IAP or the development of IAH.

#### 3.1.2. Severe burn patients

There are nine prospective studies in 434 burn patients [18,19,20,21,22,23,24,25,26], six retrospective studies in 3171 burn patients [27,28,29,30,31,32], two case reports in adults [33,34], and three case reports in children [35,36,37] investigating the relationship between fluid resuscitation and ACS (Supplementary Table S1).

##### Prevalence

The reported prevalence of IAH ranged from 57.8% to 82.6% among patients with ≥20% total body surface area (TBSA) burned. Six of the nine studies reported ACS rates between 5.5 and 28.6% [18,19,20,21,22,23,24,25,26].

##### Resuscitation Fluids & Risk Factors

A capillary leak is common in critically ill patients and leads to interstitial edema. This can be a particular problem in burn patients who require large volumes of intravenous fluid resuscitation. There is a significant correlation between IAP and resuscitation volume [22,33,34]. A volume administration of > 250 mL/kg in the first 24 h is a risk factor for ACS, and this amount is known as the Ivy index [19]. Hypertonic lactated saline (HLS) resuscitation may reduce the risk of developing secondary ACS and is associated with a lower fluid volume when compared to Ringer’s lactate (LR) solution [21,120]. Compared to colloid resuscitation, crystalloid resuscitation resulted in higher volumes of fluid per kilogram body weight, both in the first 24 h and during resuscitation with a significantly greater increase in IAP [23]. The implementation of 5% albumin in the first 24 h of resuscitation showed a trend towards less intravenous fluid. However, this did not translate into differences in the overall incidence of ACS, but it did improve outcomes [32]. Risk factors identified for acute kidney injury were IAH and the use of glycopeptides, vasopressors, and mechanical ventilation. Acute kidney injury was associated with increased 30-day mortality [25].

##### Management

Bladder pressure measurements should be performed after infusion of more than 25 mL during the acute resuscitation phase [18]. While IAH usually responds to medical therapy, the presence of ACS warrants escharotomy or surgical decompression of the abdominal cavity [18]. Non-resolution of IAH is related to a worse outcome [26,29,38].

##### Outcome

Mortality rates in the prospective studies varied from 18% to 82.6% [18,19,20,21,22,23,24,25,26]. The implementation of burn resuscitation guidelines can significantly lower mortality rates [18].

#### 3.1.3. Severe acute pancreatitis

Severe acute pancreatitis (SAP) is a disease with a 30% mortality rate and is characterized by a systemic inflammatory response, pancreatic necrosis, and multiple organ failure [40]. Appropriate early fluid resuscitation is essential to prevent complications. Three RCTs [40,41,43], one observational study [42] (total of 295 patients), one retrospective study [44], and two case reports [45,46], investigated the relationship between intravenous fluids and IAH in SAP (Supplementary Table S2).

##### Prevalence

The incidence of ACS is lower when controlled fluid resuscitation is applied [40]. This was shown in an RCT where the incidence of ACS was 72.2% in the rapid fluid expansion group versus 32.5% in the controlled fluid expansion group [43].

##### Resuscitation Fluid and Risk Factors

The type of intravenous fluid used is important in the prevention of IAH. Resuscitation with colloids resulted in less IAH compared with crystalloids [41]. Using a combination of 0.9% saline, colloids, and glutamine is possibly a more efficient resuscitation strategy for SAP (by relieving inflammation and maintaining the intestinal barrier) than 0.9% saline [40]. Significant risk factors for the development of IAH in patients with SAP include the first 24-h fluid balance, number of fluid collections (which is included in the definitive Balthazar’s CT score for severity stratification in acute pancreatitis), and serum calcium level [42].

##### Management

Early management of patients with SAP includes the initiation of CVVH to facilitate achieving a negative fluid balance and a subsequent reduction in IAH [44]. Abdominal decompression in patients with ACS may lead to a reversal of MOF [45,46].

##### Outcome

IAH is associated with a poor prognosis and an increased need for surgical interventions with associated morbidity and mortality. The reported mortality rate varied between 7.3% to 31.6% [41].

#### 3.1.4. Trauma patients

Trauma patients frequently pose a fluid resuscitation challenge since they often require rapid intravenous fluid administration to treat hypovolemia. This may include red cell concentrate (RCC) and platelets. Rapid fluid administration, together with reperfusion injury and activation of inflammatory mediators, leads to increased capillary permeability and an increased risk of developing IAH and ACS [120,121]. There are seven prospective studies investigating the relationship between intravenous fluids and IAH in 1329 trauma patients [47,48,49,50,51,52,74], fourteen retrospective studies in 4233 trauma patients [38,39,53,54,55,56,57,58,59,60,61,62,69,75], five case reports [63,64,65,66,73], and five case reports in children [37,67,68,70,71] (Supplementary Table S3).

##### Prevalence

The reported prevalence of ACS in the prospective studies varied between 8% and 36% (with a mean Injury Severity Score (ISS) range of 13–35) [47,48,49,50,51,52]. In a retrospective study by Zaydfudim et al., the implementation of a trauma exsanguination protocol significantly reduced ACS from 20% to zero [69]. Balogh found that the implementation of a standard resuscitation compared to a supranormal resuscitation reduced the incidence of IAH (20 vs. 42%) and ACS (8 vs. 16%) [53].

##### Resuscitation Fluid and Risk Factors

Trauma patients that develop ACS, as a complication of massive volume loading, receive significantly more crystalloids and blood products [48]. Aggressive crystalloid resuscitation should be minimized in severely injured patients. Neal et al., found that patients requiring massive transfusions (crystalloid resuscitation in a ratio greater than 1.5:1 per unit of RCC) were associated with a higher risk of MOF, ARDS, and ACS [50]. Although massive transfusion is associated with more complications, when blood products are delivered in a 3:2 ratio of RCC: FFP (red blood cells: fresh frozen plasma) and 5:1 for RCC: platelets, it is associated with a reduction in MOF and infectious complications, as well as an increase in ventilator-free days [63].

##### Management

Bladder pressures should be checked routinely when resuscitation volumes approach 10 L of crystalloid or ten units of packed red cells [60]. Following the resuscitation phase, fluid removal with diuretics or CVVH may restore euvolemia and may reduce IAP leading to improvement of organ failure [5,72,76].

##### Outcome

Trauma patients with ACS have more complications, mechanical ventilation, organ failure, and a longer length of stay. Mortality for this group varies between 6% and 54% [47,48,49,50,51,52,120]. Limiting crystalloids during resuscitation in trauma patients was associated with better outcomes and almost eliminated ACS [57,58].

#### 3.1.5. Medical patients

Three prospective studies (188 patients) [85,86,87], two retrospective studies (143 patients) [78,79], three case reports in adult patients [72,76,77], two prospective trials (88 patients) in children [81,84], and four case reports in children [70,80,82,83] discuss fluid resuscitation in patients with sepsis (Supplementary Table S4).

##### Incidence

The observed incidence of IAH varied between 20 and 85%, and ACS developed in 25–28% of cases [78,85]. The incidence of IAH and ACS in a group of 40 medical ICU patients with a positive fluid balance of more than 5 L/24 h was high, with 85% developing IAH and 25% developing ACS [86].

##### Resuscitation Fluid and Management

In a prospective trial of 68 children, the replacement of crystalloid fluid resuscitation with albumin for refractory shock resulted in a smaller positive fluid balance, decreased morbidity, and improved outcomes [84]. Treatment (PAL therapy) that combined high levels of positive end-expiratory pressure (PEEP), small volume resuscitation with hyperoncotic 20% albumin (up to serum albumin levels of 30 g/L), and fluid removal using furosemide (a bolus of 1 mg/kg followed by continuous infusion at 10 mg/hour and titrated according to urine output) or renal replacement therapy with net ultrafiltration was associated with a reduction of extravascular lung water index (EVLWI) and IAP, was associated with improved clinical outcomes (better survival and faster weaning from mechanical ventilation) [87].

Decompressive laparotomy (open abdomen with silo bag) has been previously successful in medical patients [5,76]. Fluid removal with diuretics or CVVH may restore fluid balance and may reduce IAP, leading to improvement of organ failure [72].

##### Outcome

ACS is associated with a high mortality rate (52.8–77.4%) [78]. Moreover, Cordemans et al., concluded that there is a correlation between poor outcomes and a high capillary leak index (CLI), a positive fluid balance, high IAPs, high extravascular lung water indices (EVLWI), and low abdominal perfusion pressures (APP) [78]. The ACS-associated mortality rate in children was 16% [81].

#### 3.1.6. Surgical patients

Six prospective studies (460 surgical patients) [92,93,94,95,96,97], four retrospective studies (189 patients) [88,89,98,99] and seven case reports (see Table 3) in adults [63,90,91,100,101,102,103], describe the association between fluid and ACS (Supplementary Table S4).

##### Incidence

Dalfino et al., showed how a positive fluid balance comprised one of three independent predictors for developing IAH (31.8%), together with baseline IAP and central venous pressure [104].

##### Resuscitation Fluid and Risk Factors

There is a significant positive correlation between increased IAP with a positive fluid balance and decreased IAP with a negative fluid balance [97]. A liberal fluid strategy, compared to a restrictive fluid strategy, is associated with a significantly higher rise in IAP after surgery [94]. Furthermore, there was a strong correlation between IAP and extracellular water content in the liberal subgroup, which is in keeping with the hypothesis of fluid extravasation being one of the critical mechanisms in the development of IAH.

Makar et al., conducted an observational study in patients following open and endovascular repair of ruptured abdominal aortic aneurysms (rAAA). The results suggested that endovascular repair is associated with less intra-abdominal hypertension and host inflammatory response, less blood loss, blood transfusion, and total intraoperative intravenous fluid infusion compared to open repair [95]. In 25 patients with rAAA who underwent emergency EVAR [88], hypotension on arrival, transfusion of three or more units of red cell concentrate, and postoperative anemia were all significantly associated with the development of postoperative ACS.

##### Outcome

Patients with high IAP have more frequent renal failure, delayed postsurgical weaning from mechanical ventilation, and worse outcomes [92]. Reported mortality among patients with IAH was 53% [93]. The development of ACS after the repair of ruptured abdominal aortic aneurysms (rAAAs) results in increased mortality, especially in patients treated by endovascular aortic repair (EVAR) [89]. Intraoperative fluid requirements were significantly higher in EVAR patients who developed ACS than those without ACS. Furthermore, Leclerc et al., showed that in patients who underwent rAAA repair, patients with ACS appeared to have higher mortality [98]. For a positive prediction, they required three of the following eight factors: anemia, prolonged shock, preoperative cardiac arrest, body mass index >30 kg/m^2^, massive fluid resuscitation and transfusions, severe hypothermia, and acidosis.

#### 3.1.7. Mixed ICU patients

Twelve prospective studies (see Table 1) [5,104,107,108,109,110,111,112,113,114,115,116] (4213 patients) and 2 retrospective studies (71 patients) [105,106] describe fluid resuscitation in medical-surgical patients.

##### Incidence

The incidence of ACS varied between 2% and 12.9% [105,106]. The incidence of IAH is 25–30% on admission and 50% after the first week of ICU stay [115].

##### Independent Predictors for IAH

Fluid resuscitation and positive fluid balance are independent predictors for IAH [108]. Body mass index is significantly associated with the development of IAH [109]. Elevated vascular permeability due to a stress-related inflammatory response is associated with a positive fluid balance. It leads to extravascular fluid accumulation, which is likely to result in gastrointestinal tract edema and increased IAP [5].

##### Outcome

Mortality rates for IAH vary from 3 to 80% [110]. The grade of IAH is inversely related to outcome [111]. Biffl et al., showed that medical patients with ACS have a 100% mortality vs. 38% in trauma patients [104]. Similar results were seen in a retrospective (see Table 2) study that showed no significant differences in fluid resuscitation and bladder pressures between groups. However, there was a significantly higher incidence of MOF and a trend towards higher mortality in medical ACS [106]. Finally, the summary of findings of pediatric studies is presented in Table 4.

**Table 1:** Summary of findings of prospective studies on fluid administration and IAH.

Author	Year	Type of Study	Patients	Resuscitation Fluids	IAP (mmHg)	Results
*Severe burn patients*						
Ivy et al. [19]	2000	Prospective	n = 10 (7 IAH; 2 ACS)	IAH: volume of fluid 9 to 35 L 579 ACS: volume of fluid 33 to 48 L	IAP: 9 to 44	2 DL; 2 patients died
O’Mara et al. [23]	2005	Observational	Crystalloid (n = 15) vs. Plasma (n = 16)	561 mL/kg crystalloid 360 mL/kg plasma	Crystalloid: 32.5 Plasma: 16.4	Crystalloid group: - ↑ resuscitation volume/kg - ↑ IAH - ↑ end-organ damage
Oda et al. [21]	2006	Observational	HLS (n = 14) LR (n = 22) (≥40% TBSA)	Needed to maintain UO: HLS 3.1 ± 0.9 mL/24 h/kg/% TBSA vs. LR 5.2 ± 1.2 mL24 h/kg/% TBSA	HLS 14% vs. LR 50% developed IAH	HLS resuscitation can reduce risk of secondary ACS with lower fluid load than LR solution
Oda et al. [22]	2006	Observational	n = 48	ACS patients received 398.7 ± 105.5 mL/kg fluid the first 24 h after injury	IAP (49 ± 12 cm H_2_O) ACS: n = 8	>300 mL/kg/24 h fluid resuscitation → ACS
Ennis et al. [18]	2008	Prospective	n = 56 BRG group n = 62 control group	>250 mL/kg volume in the first 24 h	Not reported	ACS and mortality significantly lower in BRG group (*p* = 0.03)
Ruiz-Castilla et al. [24]	2014	Observational	n = 25 (>20% TBSA)	10473 mL in pts with IAH vs. 4100 mL in no IAH (*p* = 0.03)	13 vs. 10	IAH pts: - IV fluid - organ failure - >extension of % TBSA
Wise et al. [26]	2016	Observational	n = 56	ACS 13.6 ± 16 L vs. No ACS 7.6 ± 4.1 L	IAH: n = 44 ACS: n = 16	Non-survivors: - ↑ incidence of IAH - ↑ total fluid intake - ↑ daily and cumulative fluid balance
Mbiine et al. [20]	2017	Observational	n = 64 (adults and children)	IAH in fluid overloaded patients: 16 vs. 13 IAH in patients not fluid overloaded: 10 vs. 9	Prevalence IAH: 57.8% 3.3 times increased risk of mortality with IAH Mortality with IAH: 82.6%	More IAH among the fluid overloaded patients, but difference not significant, probably due to small sample size
Talizin et al. [25]	2018	Prospective	n = 46 (38 IAH; 8 no IAH)	48 h fluid balance: With IAH: 5370 (3857–8828) mL Without IAH: 3894 (2411–5946) mL (*p* = 0.091)	Not applicable	IAH was associated with↑ mortality rate: 24 IAH + vs. 1 IAH – *p* = 0.016
*Severe acute pancreatitis*						
Mao et al. [43]	2009	RCT	n = 76	Amount of crystalloid and colloid on admission day (mL): - Group I (rapid fluid expansion): - 4028 ± 1980 and 1336 ± 816 - Group II (controlled fluid expansion): - 2472 ± 1871 and 970 ± 633 Total amount of fluid sequestration within 4 days (mL): - Group I: 5378 ± 2751 - Group II: 4215 ± 1998	Incidence of ACS 72.2% in group I vs. 32.5% in group II	Total amount of fluid sequestration, rate of mechanical ventilation, incidence of ACS and mortality were significantly higher in group I
Du et al. [41]	2011	RCT	HES = 20 RL = 21	Total infusion volumes not significantly different between 2 groups	HES: 11.25 ± 2.35 RL: 17.08 ± 4.98	HES group (*p* < 0.05): IAP lower; more urine production, earlier negative fluid balance and fewer patients received mechanical ventilation
Ke et al. [42]	2012	Observational	n = 58	24 h fluid balance: IAH: 503 (373–1431) mL No IAH 74 (−31–409) mL	Median max IAP 13.1 mmHg 36 patients developed IAH 7 patients developed ACS	Risk factors for IAH include 24 h positive fluid balance (first day), number of fluid collections, and serum calcium level
Zhao et al. [40]	2013	RCT	n = 120	NS: 61.79 ± 7.61 mL/kg/day SH (NS + HES): 46.93 ± 12.38 mL/kg/day SHG (SH + glutamine) 44.75 ± 8.53) mL/kg/day (*p* < 0.05)	IAP in NS significant higher	Compared to the NS group: patients in the SH and SHG groups: - accessed the endpoint more quickly with less fluid volume (67.26 ± 28.53 mL/kg/d, 61.79 ± 27.61 mL/kg per day vs. 85.23 ± 21.27 mL/kg per day, *p* < 0.05). - incidence of renal dysfunction, ARDS, MODS and ACS lower
*Trauma*						
Raeburn et al. [51]	2001	Observational	n = 77	28 patients with ACS vs. 49 patients no ACS	Mortality ACS 43% vs. no ACS 22% (*p* = 0.002)	- 24 h IV fluid volume not predictive for development of ACS - Patients with ACS: >complication; LOS, MV, OF
Balogh et al. [47]	2002	Prospective	n = 128 total n = 11 ACS	26 ± 2 U RCC 38 ± 3 L crystalloid	Mortality ACS: 54%	
Balogh et al. [48]	2003	Observational	n = 188	Amount of crystalloid (L) received in: - Emergency Department: primary 4 ± 1 vs. 7 ± 1 in secondary ACS - Pre-ICU: primary 8 ± 1; vs. 12 ± 1 in secondary ACS Amount of RCC (U) received in: - Emergency Department: primary 2 ± 1 vs. 6 ± 1 in secondary ACS	ACS: Primary 11 patients vs. Secondary 15 patients Mortality ACS (prim 64% vs. sec 53% vs. no ACS 17%	- ACS patients received > crystalloid and RCC vs. non-ACS pts - Higher mortality, MOF, MV in ACS - Administration of > 3 L crystalloid in Emergency Department predicts both primary and secondary ACS. - Administration of > 7.5 L of crystalloid before ICU predicts secondary ACS
Cotton et al. [74]	2009	Prospective	Pre-TEP: n = 141 TEP: n = 125	Blood products intraoperative: - TEP 14.7 U vs. pre-TEP 11 U, (*p* = 0.001) Crystalloid intraoperative: - TEP 4.8 vs. pre-TEP 7 L (*p* < 0.001) Blood products postoperative: - TEP 31 U vs. 39 U (*p* = 0.05) ACS: 9.9% in pre-TEP vs. 0% in TEP (*p* < 0.001)	Higher 30-day survival in TEP 56.8% vs. 37.6% pre-TEP (*p* = 0.001)	- Lower LOS in TEP: 12 days vs. 16 days (*p* = 0.049) - Lower ventilation days in TEP: 5.7 days vs. 8.2 days (*p* = 0.017)
Neal et al. [50]	2012	Multi-centre, Prospective	n = 452	- Median crystalloid in first 24 h post-injury > 17 L - Median blood transfusion in the first 24 h post-injury <16 U	Overall mortality 22.6%	Patients with a ratio > 1.5:1 Crystalloid: RCC have 70% higher risk of MOF and 2-fold higher risk of ARDS and ACS
Mahmood et al. [49]	2014	Observational	n = 117 DL = 102 No DL = 15	Crystalloid (L): - DL 6 ± 3 vs. - no DL 8 ± 5 (*p* = 0.02) Blood (U): - DL 5 ± 3 vs. - no DL 6.3 ± 5 (*p* = 0.02)	16.7% developed IAP > 20 mmHg in DL Mortality: 6% in DL vs. 20% in no DL *p* = 0.05	Blood transfusion and IV fluids significant correlation with IAP >20 mmHg and more metabolic acidosis
Vatankhah et al. [52]	2018	Observational	n = 100 28 ACS vs. 72 no ACS	Crystalloid: - ACS 6107 mL vs. no ACS 4493 mL RCC: - ACS 965 mL vs. no ACS 207.5 mL FFP: - ACS 1390 mL vs. no ACS 700 mL Platelets: - ACS 310 mL vs. no ACS 3.5 mL	21% mortality in ACS	Mean volume of fluids significantly higher in pts. with ACS
*Medical*						
Daugherty et al. [86]	2007	Observational	n = 40	Positive fluid balance > 5 L/24 h	n = 34 IAP > 12 mmHg n = 13 IAP > 20 mmHg n = 10 ACS	25% of patients with 5 L or > positive fluid balance in 24 h developed ACS
Cordemans et al. [87]	2012	Observational	n = 123	Cumulative fluid balance: - No IAH: 5943 ± 7125 mL - IAH: 10176 ± 7523 mL (*p* = 0.024)	20% IAH	Not achieving CLFM & being non-responder: strong independent predictors of mortality
Dorigatti et al. [85]	2019	Observational	n = 25	Accumulated fluid balance (mL): n = 13 (death): 15165.4 ± 12719.2 vs. n = 12 (survival): 6194.5 ± 6517.1	IAP 14.1 ± 4.2 vs. 9.4 ± 2.0	Higher admission and consecutive SOFA score of > 7 associated with higher ACS incidence and higher mortality rate.
*Surgical*						
Biancofiore et al. [92]	2003	Observational	n = 34 IAH n = 74 no IAH	IAH: - 5420 ± 1073 mL/d NO IAH: - 2852 ± 905 mL/d (*p* < 0.01)	Not Reported	High IAP pressure: - more frequently associated RF - delayed postsurgical weaning from MV, - worse outcome
Šerpytis et al. [97]	2008	Observational	n = 77	Not reported	POD 1: 45.5% IAH POD 2: 41.7% IAH POD 3: 35.6% IAH	Positive correlation between 24-h fluid balance and IAP
Makar et al. [95]	2009	Prospective	n = 14 eEVR n = 16 eOR	Units RCC: (*p* ≤ 0.001) - eEVR: 3 (2–4) - eOR: 9 (5–11) Intra-op IV fluid: (*p* = 0.001) - eEVR: 2250 (1500–3125) mL - eOR: 4250 (3123–7500) mL	1 ACS in eEVR, 1 ACS eOR	Correlation between IAP and the following: - blood loss and transfusion - fluid - SIRS - MOD - LOS ICU and hospital
Dalfino et al. [93]	2013	Observational	n = 22 IAH n = 47 no IAH	Positive fluid balance: independent risk factor for IAH	Not Reported	Mortality IAH 53% vs. 27% (*p* = 0.02)
Muturi et al. [96]	2017	Observational	n = 113	IV fluid over 24 h (mL): IAH: 3946.6 vs. No IAH: 2931.1 (*p* = 0.003)	n = 76 IAH n = 37 no IAH n = 5 ACS	Of those who had IAH; age, amount of iv fluids over 24 h, fluid balance & ventilator mode were significant determinants of risk of progression to ACS
Kotlińska-Hasiec et al. [94]	2017	Observational	Liberal: n = 32 vs. Restrictive: n = 31)	Liberal = 2822 ± 606 mL Restrictive = 823 ± 223 mL (*p* < 0.001)	Significant higher IAP in pts receiving liberal crystalloid therapy	Correlation between IAP and ECW
*Medical-surgical*						
Biffl et al. [104]	2001	Prospective	14 ACS: 8 trauma 6 medical	Averages administered: 16.7 ± 3.0 L crystalloid 13.3 ± 2.9 RBC	10 patients underwent DL	- 38% mortality in trauma - 100% mortality in medical patients
Malbrain et al. [110]	2004	Observational	n = 97	Patients with IAH: - higher rate of fluid resuscitation; odds ratio 3.3 (95%CI 1.2–9.2) - more frequently transfused; odds ratio 7.3 (95%CI 0.9–60.3)	IAH 50.5% ACS 8.2%	- Fluid resuscitation at limit of statistical significance as predictor of IAH - BMI significantly associated with IAH
Malbrain et al. [109]	2005	Observational	n = 265	Not reported	IAH: 32.1% on admission Mortality 27.5%	Fluid resuscitation was independent predictor for IAH (OR, 1.88; 95% CI, 1.04– 3.42; *p* = 0.04)
Dalfino et al. [107]	2008	Observational	n = 123	Cumulative fluid balance in ml - IAH: 3760 ± 4500 mL - No IAH: 680 ± 3040 mL *p* = 0.001	Primary IAH: 27.1% Secondary IAH: 67.5% ACS: 5.4%	Acute renal failure: 19.7% in IAH vs. 8.1% in no IAH Age, cumulative fluid balance and shock are all independent predictive factors of IAH
Vidal et al. [113]	2008	Prospective	n = 83	Intense fluid resuscitation, was significantly greater in patients with IAH and in non-survivors	53 patients with IAH 30 patients with no IAH	IAH associated with organ failure and mortality
Reintam Blaser et al. [116]	2011	Observational	n = 563	>5 L fluid resuscitation/24 h: - No IAH: 163 patients - IAH: 100 patients *p* = 0.009	No IAH: 381 patients IAH: 182 patients 33% mortality in ACS patients	- BMI > 30, PEEP > 10, P/F < 300, vasopressors, inotropes, pancreatitis, hepatic failure/cirrhosis and ascites, GI bleeding, laparotomy on admission are all independent risk factors for IAH
Kim et al. [114]	2012	Observational	n = 100 52 medical, 37 surgical, 11 trauma	No correlation with 24-h fluid balance	42% IAH, 4% ACS	- BMI > 30, high CVP, infection and sepsis associated with IAH - There was a 16% mortality
Iyer et al. [111]	2014	Observational	n = 403	IAH: 4.24 (2.54–5.56) L No IAH: 2.75 (1.75–4.05) L (*p* < 0.001)	39% IAH 2% ACS	IV fluid > 2.3 L is an independent predictor of IAH
Malbrain et al. [115]	2014	Systematic review	n = 1669	Not reported	Overall mean IAP: - 9.9 ± 5 mmHg - 27.7% pts IAH: - mean IAP 16.3 ± 3.4 mmHg - 30.8% died in ICU	- Independent predictors for IAH: SOFA score and fluid balance on admission - Independent predictors for ICU mortality: IAH, SAPS II, SOFA
Dąbrowski et al. [5]	2015	Observational	n = 120 48 surgical 72 medical	Cut-off points for development of IAH: - Medical: 22.4 L of ECW; 6.6 L of Volume excess - Surgical: 24.9 L of ECW; 9.5 L of Volume excess	Not Reported	IAP strongly correlates with ECW
Murphy et al. [108]	2018	Observational	n = 285	No IAH: 1135 (145–2685) mL IAH: 2019 (716–4.000) mL (*p* < 0.001)	45% IAH, 3% ACS Mortality: 30% IAH vs. 11% no IAH	24-h fluid balance > 3 L is an independent predictor for IAH
Reintam Blaser et al. [112]	2019	Observational	n = 491	48.9% IAH (46.3% primary vs. 53.7% secondary). IAH vs. no IAH: 5 L fluid resuscitation before ICU (*p* = 0.036)	6.3% ACS	Positive fluid balance and BMI and PEEP ≥ 7 cmH_2_O associated with development of IAH

DL: damage control laparotomy; UO: urine output; IV: intravenous; HLS: hypertonic lactated saline; TBSA: total body surface area; OF: organ failure; SAPS II: Simplified Acute Physiology Score II; SOFA: Sequential Organ Failure Assessment Score; ICU: intensive care unit; PEEP: positive end expiratory pressure; BRG: burn resuscitation guidelines, TEP: Trauma Exsanguination Protocol; RF: risk factor; CLFM: conservative late fluid management; 0.9% saline (NS group), combination of 0.9% saline and hydroxyethyl starch (HES) (SH group), combination of 0.9% saline, hydroxyethyl starch and glutamine (SHG group); L: liters; P: platelets; LOS: length of stay; MV: mechanical ventilation; OF: organ failure; ECW: extracellular body water; eEVR: emergency endovascular repair; eOR: emergency open repair; POD: post-operative day.

**Table 2:** Summary of findings of retrospective studies on fluid administration and IAH.

Author	Year	Population	Patients	Resuscitation Fluids	IAP (mmHg)	Intervention	Results
Boehm et al. [27]	2019	Burn	38 ACS+ vs. control	Average FB/day: ACS vs. control 13.3 L ± 7.7 L vs. control 7.9 L ± 7.9 L (NS)	Not reported	Not reported	↑ mortality rate of 84% in ACS+ vs. 32% in control (*p* = 0.00008)
Hershberger et al. [28]	2007	Burn	25 ACS+	Mean fluid infused 2102 mL/h before DL	Mean IAP 57 ± 4.2	DL	22 patients (88%) died
Hobsen et al. [29]	2002	Burn	n = 1014 10 ACS	3.1 mL/kg/% TBSA for the first 12 h	Mean 40 ± 10	DL	40% of ACS patients survived
Markell et al. [30]	2009	Burn	n = 1825 ACS: 32	6.02 mL/kg/% TBSA	>30	DL	90% mortality for ACS
McBeth et al. [31]	2014	Burn	110	48-h FB: 25.6 (± 11.1) L exceeding predicted Parkland formula estimates by 86% (± 32)	12.1 (± 4.2)	3 patients DL	39 patients died
Park et al. [32]	2012	Burn	159	Pre-protocol 4.6 ± 2.3 mL/kg/% TBS. Post-protocol: 4.2 ± 1.7 mL/kg/% TBS, mean ± SD; *p* not significant	Pre-protocol: - 10% ACS Post-protocol: - 2% ACS *p* not significant	DL, n (%) Pre-protocol: 6 Post-protocol: 0 (*p* < 0.05)	Mortality, n (%) - 26 (26) vs. 5 (10) - (*p* < 0.01)
Britt et al. [38]	2005	Burn, trauma	10 ACS	Mean volume in the first 24 h: 33 L (12.4–69)	Mean 44.6	DL	- Overall mortality 60% Mortality in DL: 43%
Reed et al. [39]	2006	Trauma, burn, solid organ injury	12	12 L of fluids or >500 mL/h for 4 consecutive hours	Average before and after catheter insertion 44.8 and 58.7	2 patients DL, 8 patients intra-abdominal catheters	7 patients survived
Gracias et al. [54]	2002	Trauma	5 ACS vs. 15 control	ACS: 37 L crystalloid vs. Control: 16.1 L crystalloid	>25	Decompression	60% in ACS vs. 7% in control
Balogh et al. [53]	2003	Trauma	71 N vs. 85 SN	SN vs. LR infusion: - mean ± SD 13 ± 2 L vs. 7 ± 1 L (*p* < 0.05)	SN vs. LR: - IAH 42% vs. 20% (*p* < 0.05) - ACS 16% vs. 8% (*p* < 0.05)	Not reported	Mortality SN vs. LR: 27% vs. 11% (*p* < 0.05)
He et al. [55]	2019	Trauma	455 pts (44 IAH; 5 ACS)	Volume of IV fluids over 24 h: 3.965 ± 739 mL	Mean IAP 24.4 ± 8.5	DL	- Mortality in DL 15% - Mortality in ACS 40%
Hwabejire et al. [56]	2016	Trauma	n = 1976 of which 122 ACS	Total fluid/kg: - ACS+ 498 ± 268 mL/kg vs. ACS- 293 ± 171 mL/kg (*p* < 0.001)	Not reported	98.4% DL	ACS+: 37.7% vs. ACS-: 14.6% (*p* < 0.001) Rise in ACS risk after total volume + 1302 mL/kg
Joseph et al. [57]	2014	Trauma	799	- DL in 151 patients - Mean crystalloids in ACS after DL 23 L - 4 patients with ACS after DL	18 patients ACS	DL in 18.9%	- Overall mortality: 14.5% - ACS mortality: 55.6% - DL mortality: 47%
Macedo et al. [58]	2016	Trauma	10	- Average crystalloid intraoperatively: 12.8 ± 8.2 L (range 3–30 L) - Mean U RCC: 25.6 ± 16.31 U (9–53) - Mean U FFP 13.5 ± 10.6 U (4–36) - Mean U platelets:11.5 ± 9.4 U (0–30)	Not reported	DL	60% overall mortality
Shaheen et al. [62]	2016	Trauma	28	>10 U of RCC in 24 h	60.7% developed ACS	Not reported	- 30-day mortality was 32.1%
Madigan et al. [59]	2008	Trauma	ACS (n = 48) vs. control (n = 48)	Net fluid for DC until 48 h post-admission was 18.2 L vs. 5.1 L (*p* < 0.0001)	Not reported	DL	Mortality 60% ACS vs. 2% controls (*p* < 0.0001)
Maxwell et al. [60]	1999	Trauma	46	Mean 19 ± 5 L crystalloid 29 ± 10 U RCC	Mean: 33± 3	DL	67% mortality
Rodas et al. [61]	2005	Trauma	5	Crystalloid: 15 ± 1.7 L Blood: 11 ± 0.4 U	NR	DL	No mortality
Strang et al. [75]	2015	Trauma	567 509 no IAH 58 IAH	No IAH: 4.2 L Crystalloid vs. IAH: 6 L crystalloid; no IAH: 1.5 L colloids vs. IAH: 2.5 L colloids; no IAH: 2 U RCC vs. IAH: 17 U	30 patients ACS	NR	IAH: 25.9% vs. 12.2% no IAH; *p* = 0.012).
Zaydfudim et al. [69]	2010	Trauma	39 pre-TEP vs. 36 TEP	Pre-TEP: 12 U RCC vs. TEP: 12.5 U RCC Pre-TEP: 4 U FFP, vs. TEP: 8 U FFP; *p* < 0.01 Pre-TEP: 1 U platelets vs. TEP: 2 U platelets; *p* < 0.01 Pre-TEP: 6 L of crystalloids vs. TEP: 4 L crystalloids; *p* < 0.01	20% ACS in pre-TEP vs. 0% ACS in TEP	NR	pre-TEP cohort: 31% 30-day survival TEP cohort: 53% 30-day survival
Cothren et al. [106]	2007	Surgical & Medical patients	54 patients	Total fluid resuscitation before DL: - Medical patients: 18.5 ± 1.8 L vs. - Surgical patients: 16 ± 1.5 L (NS) Total transfusion of RCC: Medical: 3.7 ± 1.8 U vs. Surgical: 14.5 ± 2 U (*p* = 0.006)	Medical: 33.5 ± 1.1 vs. - Surgical: 32.8 ± 1.8	DL	MOF: - Medical patients: 62% vs. Surgical patients: 27% (*p* < 0.05) Mortality: - Medical patients: 54% vs. Surgical patients: 34%
Cordemans et al. [78]	2012	ALI	57 PAL vs. 57 control	Cumulative FB after 1 week 8.027 ± 5.254 mL/day vs. −1.451 ± 7.761 (*p* < 0.001)	IAP at baseline: PAL: 10 ± 4.2 Control: 8 ± 3.7 (*p* = 0.013)	PAL treatment	- Overall mortality 38.6% (n = 44) - 49.1% in control vs. 28.1% PAL (*p* = 0.034)
Pupelis et al. [44]	2012	Pancreatitis	130 patients 75 CVVH 55 control	Not reported	CVVH: 19.6 ± 7.1 Control: 16.3 ± 5.5 *p* = 0.05	DL n = 36	11.7% CVVH and 13.8% no CVVH NS
Struck et al. [79]	2012	TEN	29 patients 5 ACS	+ FB 4.6 ± 1.2 L	33 ± 7	DL	Mortality: ACS+ 100% vs. ACS- 0%
Aik-Yong et al. [105]	2014	Surgical & medical patients	17 patients: 14 primary ACS 3 secondary ACS	>3.5 L in 24 h		DL	Overall mortality 47.1%
McNelis et al. [99]	2002	Surgery	22 ACS vs. 22 control	24-h FB: ACS: 15.9 ± 10.3 L vs. Control: 7 ± 3.5 L (*p* < 0.05)	Not reported	Not reported	Mortality: 66.7% in ACS vs. none in control
Rubenstein et al. [89]	2015	rAAA open repair. 44 pts (60%) EVAR: 29 pts (40%)	73	Intraoperative fluid higher in EVAR patients ACS+ vs. ACS- - RCC: 5600 mL vs. 1100 mL (*p* < 0.0001) - Total blood products 9300 vs. 1500 mL (*p* < 0.001) - Crystalloid 11200 vs. 4500 mL (*p* < 0.001)	ACS% 34% in open21% in EVAR*p* not significant	DL	Overall mortality 42%: - 31% EVAR - 48% open repair Mortality: - ACS+: 62% vs. 33% ACS–(*p* = 0.022)
Leclerc et al. [98]	2017	rAAA	47	ACS+: 5.250 (4.625; 9.375) L ACS-: 4.125 (2.925; 5.500) L (*p* = 0.053)	8 patients developed ACS		30-day mortality in ACS+ higher (*p* = 0.108)
Miranda et al. [88]	2018	rAAA	25	- 36% received ≥ 3 U RCC preoperatively and intraoperatively. - 36% received ≥ 3 L of crystalloid. - All of those who developed ACS received more than 3 U RCC; 67% received >3 L of crystalloid	12% (n = 3) developed ACS		- Overall mortality rate: 28% - Mortality rate in ACS: 67%

FB: fluid balance; pts: patients; ACS+: with abdominal compartment syndrome; ACS-: without abdominal compartment syndrome; TBSA: total body surface area; DL: decompressive laparotomy; EVAR: endovascular aortic repair, NS: not significant; rAAA: ruptured abdominal aortic aneurysms; U: units; RCC: Red cell concentrate; PAL: peep-albumin-Lasix; CVVH: continuous veno-venous hemofiltration; S: surgical; M: medical; SN: supranormal resuscitation group; LR: lactated ringer infusion; d: day.

**Table 3:** Summary of findings of case reports on fluid administration and IAH.

Author	Year	Population	Resuscitation Fluids/Fluid Balance	IAP (mmHg)	Intervention	Results
Fietsam et al. [101]	1989	Surgery	>25 L of fluid	NR	DL	NR
Burrows et al. [63]	1995	Surgery	21 L of crystalloid; 4 U RCC	NR	DL	Alive
Burrows et al. [63]	1995	Trauma	Pre-op: 7.3 mL/kg/h vs. Postop: 14.2 mL/kg/h	39	DL	NR
Burrows et al. [63]	1995	Trauma	Pre-op: 9.2 mL/kg/h vs. Postop: 5.5 mL/kg/h	40	DL	Died
Burrows et al. [63]	1995	Trauma	Pre-op: 14.7 mL/kg/h vs. Postop: 3.2 mL/kg/h	NR	DL	Alive
Ivy et al. [33]	1999	Burn	32 L	49	DL	Died
Ivy et al. [33]	1999	Burn	24 L	50	Escharotomy	Died
Ivy et al. [33]	1999	Burn	32 L	36	None	Died
Kopelman et al. [65]	2000	Trauma	+ FB: 25 L	34	DL	Died
Kopelman et al. [65]	2000	Trauma	26 L of crystalloid	25	DL	Died
Kopelman et al. [65]	2000	Trauma	+ FB: 29.5 L	22	DL	Died
Kopelman et al. [65]	2000	Trauma	+ FB: 10 L	26	DL	Alive
Kopelman et al. [65]	2000	Trauma	+ FB: 5 L	46	DL	Alive
Macalino et al. [77]	2002	Sepsis	14 L crystalloids	27	NMB	Died
Kula et al. [72]	2004	Sepsis	10 L + FB first 96 h. 4:1 (crystalloid: colloid)	>25	DL CVVH	Died
Kula et al. [72]	2004	Sepsis	12.5 L + FB first 96 h (crystalloids)	29	CVVH	Died
Shiiya et al. [103]	2005	Surgery	34.1 L crystalloids vs. 13.7 L blood products	NR	DL	Alive
Parra et al. [34]	2006	Burn/Trauma	25.55 L of crystalloid 12 U RCC	34	DL	Alive
De Wolf et al. [100]	2008	Surgery	Massive fluid resuscitation	24 in 1st patient 27 in 2nd patient	DL	Alive
Tsuang et al. [76]	2007	Sepsis	17 L fluid during first 20 h	54	DL	Alive
Chamisa et al. [64]	2008	Trauma	Not reported	>35	DL	Died
Kula et al. [73]	2008	Trauma	7.5 L + FB first 48 h. 4:1 (crystalloid: colloid)	26	CVVH	NR
Kula et al. [73]	2008	Trauma	17 L + FB first 96 h. 3:1 (crystalloid: colloid)	28	CVVH	NR
Augustin et al. [90]	2010	Surgery	16 L + FB	19	DL	Died
Augustin et al. [90]	2010	Surgery	23 L + FB	35	None	Died
Rabbi et al. [102]	2012	Surgery	Not reported	50	DL	Alive
Park et al. [46]	2014	SAP	Not reported	31	PCD	Alive
Bressan et al. [91]	2016	Surgery	4 L crystalloids 2 RCC during first 24 h	21	DL	Alive
Michel et al. [66]	2016	Trauma	10.5 L (crystalloids, colloids & blood products)	NR	DL	Alive
Lee et al. [45]	2019	SAP	6 L	28	DL	Alive

+ FB: positive fluid balance; NR: not reported; CVVH: continuous veno-venous hemofiltration; NMB: neuromuscular blocker; SAP: severe acute pancreatitis; PCD: Percutaneous Catheter Drainage; DL: decompressive laparotomy; RCC: red cell concentrate.

**Table 4:** Summary of findings of pediatric studies on fluid administration and IAH.

Author	Year	Type of Study	Population	Resuscitation Fluids	IAP (mmHg)	Intervention	Results
Divarci et al. [81]	2016	Prospective	Sepsis	NR	14 patients with IAH (13–15) 6 patients ACS (17–24)	Decompressive measures DL	1 Dead
Ranjit et al. [84]	2018	Prospective	Sepsis	ST group (n = 30): 17.8 (10.8–25.2) L TI group (n = 38): 10.02 (5.7–18.2) L (*p* = 0.009)	NR	Percutaneous drainage of ACS, n (%) ST group: 9 (30) TI group: 3 (7.9) (*p* = 0.01)	Mortality: ST: 8 (26%) TI: 1 (2.6%) *p* = 0.008
DeCou et al. [70]	2000	Case report	Trauma	Crystalloids and 16 U RCC and 4 U FFP	NR	Silo decompression	Alive
DeCou et al. [70]	2000	Case report	Trauma	Replacement of 2 x blood volume	NR	Silo decompression	Alive
DeCou et al. [70]	2000	Case report	Sepsis	NR	26	Silo decompression	Alive
Perks et al. [68]	2005	Case report	Trauma	NR	NR	Surgical decompression	Alive
Jensen et al. [37]	2006	Case report	Burn	5990 mL crystalloids	>22	DL	Dead
Jensen et al. [37]	2006	Case report	Burn	8580 mL crystalloids + 990 mL blood products + 805 mL albumin	NR	Abdominal wall escharotomy and NMB and peritoneal dialysis catheter	Alive
Jensen et al. [37]	2006	Case report	Burn	10300 mL crystalloids	44	Surgical decompression	Dead
Jensen et al. [37]	2006	Case report	Trauma	1950 mL crystalloids	26	Silo decompression	Alive
Morell et al. [67]	2007	Case report	Trauma	10000 mL crystalloids and 10 U RCC	NR	Laparotomy	Alive
Lam et al. [83]	2008	Case report	Sepsis	272 mL/kg	35	Paracentesis	Died
Lam et al. [83]	2008	Case report	Sepsis	220 mL/kg	NR	DL	Died
Lam et al. [83]	2008	Case report	Reanimated after drowning	334 mL/kg	NR	DL	Died
Lam et al. [83]	2008	Case report	Sepsis	500 mL/kg	120	None	Died
Lam et al. [83]	2008	Case report	Sepsis	NR	NR	Peritoneal catheter	Alive
Dauplaise et al. [80]	2010	Case report	Sepsis	70 mL/kg in first h and 330 mL/kg in first 24 h	43	DL	Alive
Gala et al. [82]	2012	Case report	Sepsis	NR	NR	Paracentesis	Alive
Streit et al. [35]	2013	Case report	Burn	NR	27	Decompression	Alive
Sun et al. [36]	2015	Case report	Burn	5600 mL LR during first 24 h	22	NMB, diuresis; percutaneous drain	Alive
Kobayashi et al. [71]	2016	Case report	Trauma	560 mL RCC. 960 mL FFP. 400 mL platelets and fluids	NR	Laparotomy	Alive

NR: not reported; RCC: red cell concentrate; FFP: fresh frozen plasma; ST group: standard therapy; TI group: targeted intervention; DL: decompressive laparotomy; NMB: neuromuscular blockers.

#### 3.2. Animal data

**Figure 3:**
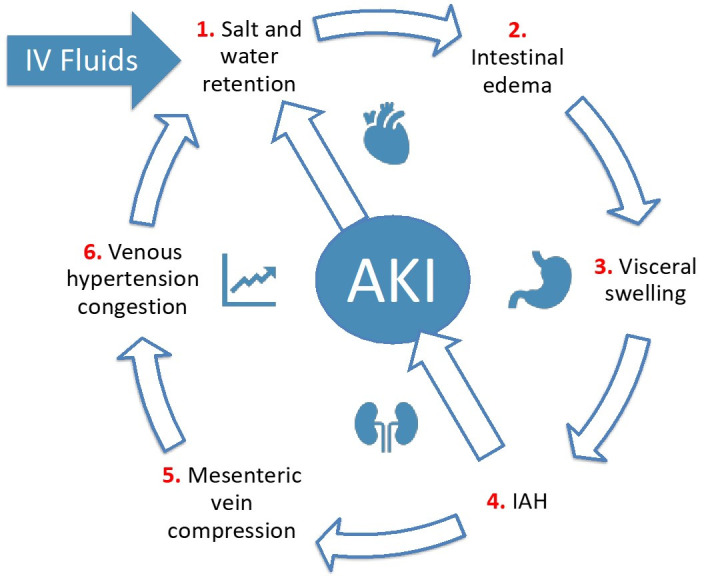
The vicious cycle of fluid resuscitation, abdominal hypertension and kidney injury. Adapted according to the Open Access CC BY License 4.0 from Malbrain et al., with permission [17]. AKI: acute kidney injury; IAH: intra-abdominal hypertension.

We found eleven animal studies, of which three were suitable, reporting on resuscitation and secondary IAH (Table 5). Fluid resuscitation leads to IAH and venous congestion (or venous hypertension), resulting in gut edema and diminished gut contractility [117]. Melatonin may prevent deleterious effects related to fluid overload [118]. Extensive fluid resuscitation preserves cardiac output, urine output, and serum parameters (e.g., ALT, lipase, AP, lactate, creatinine) in pigs with ACS, but organ damage occurs (vicious cycle) [119]. Previous animal studies showed that IAH provokes the release of pro-inflammatory cytokines which may serve as a second insult for the induction of MOF [121]. This is illustrated in Figure 3.

**Table 5:** Summary of findings of animal studies on fluid administration and IAH.

Author	Year	Population	Intervention	Results
Schachtrupp et al. [119]	2005	12 Pigs: - 6 intervention group (IAP to 30 mmHg) - 6 control group	Fluid intake: Intervention group vs. control (*p* < 0.01) 10570 ± 1928 mL vs. 3918 ± 1042 mL	Acidosis, liver, bowel, kidney and lung damage higher in intervention group (*p* < 0.01)
Moore-Olufemi et al. [117]	2005	44 Rats Experiment 1: 20 mL/kg saline Experiment 2: 80 mL/kg saline In each experiment 4 groups - no venous HTN/no resuscitation (sham, n = 6), - venous HTN/resuscitation (n = 6), - no venous HTN/resuscitation (n = 6), - venous HTN/no resuscitation (n = 4)	A mesenteric venous hypertension/gut edema model was created to evaluate whether gut edema caused by acute mesenteric venous hypertension and/or crystalloid resuscitation is associated with impaired intestinal transit, mucosal barrier dysfunction, and/or injury	Delayed intestinal transit, increased permeability, and decreased epithelial resistance are associated with gut edema
Chang et al. [118]	2016	48 rats: - Sham group (n = 8) - shock group (n = 8) - LR group (n = 8) - melatonin group and LR (n = 8) - HS + LR group (n = 8) - HES + LR group (n = 8)	Induced portal hypertension, hemorrhage to a MAP of 40 mmHg for 2 h (except for sham group) Collected blood reinfused and treatment with: - LR (30 mL/h), - melatonin (50 mg/kg) + LR, - HS (6 mL/kg) + LR, - HES 30 mL/kg +LR. - shock: no fluids	Melatonin use associated with less inflammatory and oxidative injury, less intestinal permeability and injury, lower incidence of secondary IAH

LR: Ringer’s lactate solution, HES: hydroxyethyl starch, IAH: intra-abdominal hypertension.

## 4. Discussion

Existing studies and pathophysiological rationale support the association between fluid administration and IAH. However, current evidence does not allow clinicians to accurately identify specific fluid management strategies for patients with IAH. IAH often occurs in patients with sepsis, trauma, burns, and severe acute pancreatitis [122,123,124]. These conditions are united by an accompanying inflammatory response that often progresses to shock and requires ongoing intravenous fluid therapy. Addressing the underlying cause of the pathophysiological process is essential; however, in all these patients, fluid management remains a challenge. Avoiding hypovolemia as well as unnecessary excessive intravenous fluids and subsequent interstitial edema, with progression to IAH and ACS, is a difficult balance to achieve [125,126].

The origin of intravenous fluid therapy [127] dates back to the cholera outbreak in the 1830s. Resuscitation fluids are administered to restore intravascular volume and maintain tissue perfusion [17]. However, determining the volume status of a critically ill patient remains a diagnostic challenge [123]. Furthermore, the ideal synthetic intravenous resuscitation fluid does not exist. Both crystalloid and colloid solutions offer therapeutic options. Albumin is considered safe for use as a resuscitation fluid in most critically ill patients; however, in patients with traumatic brain injury, its use is associated with increased mortality [128]. The use of hydroxyethyl starch (HES) solutions is associated with increased rates of renal-replacement therapy and blood transfusion in patients with sepsis and surgery. The use of 0.9% saline has been associated with the development of metabolic acidosis and acute kidney injury.

**Figure 4:**
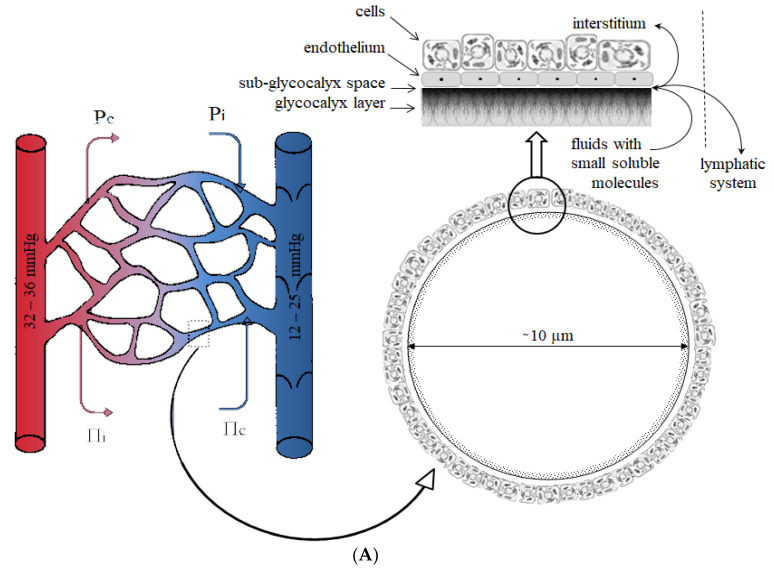

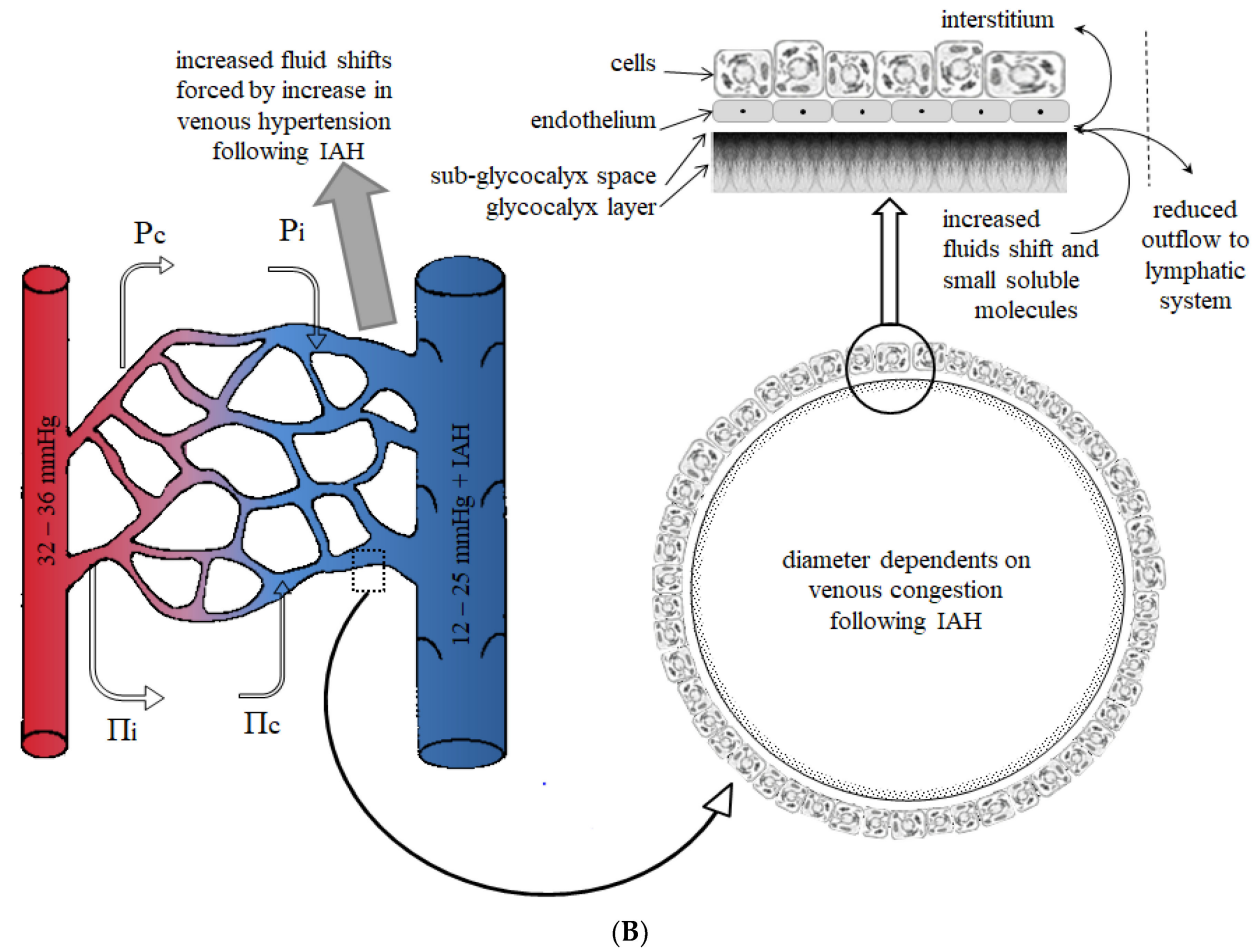
Fluid movement in normal conditions (**A**) and abdominal hypertension (**B**). The physiological movement of fluid is determined by the imbalance between hydrostatic and colloid osmotic pressures. It is best described by the revised Starling equation: *J_v_* = *L_p_A*[(*P_c_* − *P_i_*) − *σ*(*II_c_* − *II_i_*)], where *J_v_* is net fluid filtration, *L_p_* the capillary hydraulic permeability, *A* the capillary surface area (which is available for fluids and small molecule filtration), *σ* the capillary reflection coefficient, *P_c_* the capillary hydrostatic pressure, *P_i_* the interstitial hydrostatic pressure, *II_c_* and *II_i_* the capillary and interstitial colloid osmotic pressures, respectively. Generally, *P_c_* dependent on the differences between the arteriole hydrostatic pressure (*P_A_*) and the venule hydrostatic pressure (*P_V_*). This difference strongly corresponds to the hydraulic resistances in arterioles and venule (*R_A_* and *R_V_*, respectively), which was described by the Pappenheimer Soto-Riviera Equation: *P_c_* = (*P_v_* [*R_A_*/*R_V_*] + *P_A_*)/(1 + [*R_A_*/*R_V_*]). According to this equation, every increase in *P_A_* or *P_V_*, as well as an increase in *R_A_*/*R_V_* (e.g., following intra-abdominal hypertension leading to venous congestion) or increase *P_c_*. Under normal physiological conditions, the sub-glycocalyx colloid osmotic pressure strongly corresponds to interstitial pressure and its value ranges between 70% and 90% of the interstitial colloid pressure. Adapted from Levick et al. [133].

Fluid movement through the microcirculation is partly determined by the imbalance between colloid osmotic and hydrostatic forces (Starling equation). Following this theory in IAH, an increase in microvenule blood pressure following venous compression reduces the difference in hydrostatic pressure, resulting in disturbance of microcirculatory fluid movement. The entire vascular endothelium is covered by the endothelial glycocalyx which consists of various proteoglycans, glycoproteins, and glycolipids. It which plays a vital role in the movement of fluids. The endothelial glycocalyx is semi-permeable to small molecules and ions and impermeable to molecules greater than 70 kDa [129,130]. The Starling equation has been revised to account for the sub-glycocalyx layer that contributes to a reflectance coefficient responsible for larger molecules staying intravascular. According to this revised Starling equation, the differences in plasma-sub-glycocalyx colloid osmotic pressure play a crucial role in trans-endothelial fluid movement [131]. The revised Starling equation has the sub-glycocalyx oncotic pressure replacing the interstitial oncotic pressure as a primary factor in transvascular fluid movement (Figure 4). The rule states that colloids such as albumin may delay transvascular fluid escape under selected conditions but will not pull fluids from the interstitium back into the vascular compartment; rather, albumin only returns to the intravascular compartment by the lymphatics [132,133]. A decreased arterial pressure in conjunction with an increased venous pressure is frequently observed in patients with IAH. Increased pressure in venules may increase hydrostatic capillary pressure and augment transcapillary fluid extravasation causing loss of plasma volume. This is because of the dependence on differences in transendothelial pressure for the movement of fluid. Thus, the administration of colloid solutions to restore mean arterial pressure may maintain colloid osmotic pressure but increase hydrostatic capillary pressure, which may intensify fluid filtration. Crystalloid solutions decrease colloid osmotic pressure and increase hydrostatic capillary pressure, theoretically leading to higher fluid filtration than colloids [131]. However, IAH is often the result of several pathologies coinciding, damaging the glycocalyx and causing increased vascular permeability. As a result, both crystalloid and colloid solutions leak from the intravascular compartment into the interstitial space.

Experimental models have confirmed that when maintaining a normal MAP of approximately 65 mmHg (using vasopressors), fluid movement and reduction of plasma volume are more pronounced when the capillary permeability is disrupted versus normal conditions [134]. Interestingly, the plasma-reducing effect was lower in hypovolemic conditions compared to normovolemic subjects. These findings may suggest that the decrease in hydrostatic capillary pressure following hypovolemia leads to higher fluid retention in the intravascular space [135]. This effect may be disrupted by IAH; however, this hypothesis is yet to be confirmed.

### 4.1. Type of Patients

The incidence of ACS and IAH differs across various patient populations, but with a high mortality rate, regardless of the population.

In severe burns, the systemic release of inflammatory and vasoactive mediators is responsible for a systemic capillary leak, intravascular fluid loss, and significant fluid shifts that should be managed with aggressive intravenous fluid resuscitation [136]. The implementation of the Parkland formula, developed by Baxter and Shires, reduced inadequate resuscitation in acute burn patients, which in turn significantly decreased burn mortality [137]. However, excessive intravenous fluid administration during resuscitation can also be detrimental and lead to an IAH prevalence as high as 82.6% in patients with more than 20% TBSA burned. Fluid creep is applied to a burn resuscitation, during which more fluid than predicted by standard formulas is administered. Increased fluid requirements may be necessary, but dangerous fluid creep is also caused by overly permissive fluid infusion and the lack of colloid supplementation [138]. Fluid creep is reported in 30% to 90% of patients with major burns [139,140]. Complications of fluid overload include extremity and abdominal compartment syndromes, respiratory failure, and ocular hypertension [138]. Factors that predispose to increased fluid requirements are inhalation injury, delay in resuscitation, and polytrauma or high voltage electrical injury [120]. The use of hypertonic saline, 5% albumin, and routine use of a burn resuscitation guideline are all measures to help limit unnecessary fluid resuscitation.

Severe acute pancreatitis is associated with high mortality rates [141], and the local and systemic inflammatory response in SAP leads to intravascular fluid depletion and extravascular fluid accumulation, leading to IAH and ACS. Generally, in patients with IAH, volume status is probably best monitored with volumetric preload indicators instead of barometric ones (such as central venous pressure and pulmonary capillary wedge pressure) [142]. The primary aim of fluid replacement is to improve circulatory dysfunction, which leads to tissue hypoperfusion, ischemia, and self-sustaining disease with persistent pancreatic injury, extra-pancreatic tissue damage, and organ failure [143]. Although many controversies exist about the ideal fluid strategy, an RCT performed on 76 patients with SAP showed that controlled, more conservative, fluid resuscitation offers a better prognosis in patients with severe volume deficit within 72 h of SAP onset [43,144]. Initiation of renal replacement therapy should be considered to help manage fluid accumulation and ACS.

In patients with SAP, sepsis, septic shock, or severe trauma, shock-induced endotheliopathy (SHINE) is responsible for endothelial cell and glycocalyx damage [145]. Disruption of the endothelial glycocalyx layer (EGL) can also be induced by rapid infusion of intravenous fluids (partly due to the release of atrial natriuretic peptide) and acute hyperglycemia [131]. In septic patients, interstitial oncotic pressure increases due to the capillary leak, leading to a reduction of the plasma-expanding efficacy of any infused fluid [131] and aggravating the development of tissue edema. More recently, it has been suggested that non-resuscitation fluids in critically ill patients may even have a more considerable absolute impact on cumulative positive fluid balance than resuscitation fluids. In contrast, unintentional fluid administration in the form of IV medications and concentrated electrolytes contributes to the phenomenon of ‘fluid creep’ [146].

Understanding the different phases of intravenous fluid management (Figure 1 represents the ROSE concept) is key to planning optimal fluid management. Hypovolemia should generally be treated with fluids and vasoplegia with vasopressors, but this balance is difficult to find in septic patients. Early vasopressors, in addition to fluid resuscitation, instead of fluids alone, may be necessary to avoid fluid overload [17,74,75,119,122,147,148]. The recent results of the CLASSIC trial have shed more light on this topic and showed that giving less fluids is not harmful [149]. On average IAH is observed in up to 43.5% of patients with severe sepsis [150].

### 4.2. Type of Resuscitation Fluids

Crystalloid fluids are the mainstay of fluid resuscitation; however, the findings of this review suggest alternative strategies require further investigation. A randomized controlled trial (RCT) compared HES with Ringer’s lactate resuscitation in 41 patients with SAP. Resuscitation using colloids resulted in a lower IAP and reduced need for mechanical ventilation compared to those in which Ringer’s lactate was used [41]. However, there is no evidence from RCTs that resuscitation with colloids in patients with trauma, burns, or following surgery, reduces the risk of death compared to resuscitation with crystalloids [151]. There is evidence of harm from synthetic colloids, especially synthetic starch solutions [152].

Balanced crystalloids may have advantages over 0.9% saline, possibly reducing inflammation, but no apparent effect on mortality or morbidity was demonstrated in patients with SAP [153,154]. The recently conducted pragmatic SMART study (involving 15802 critically ill adults) showed that using balanced crystalloids for intravenous fluid administration resulted in a lower rate of composite outcomes, including death from any cause, new renal-replacement therapy, or persistent renal dysfunction than the use of saline [155]. Accordingly, several current guidelines suggest using balanced rather than unbalanced crystalloids in extensive volume replacements, surgical patients, and in SAP [142,154,156].

Several studies (SAFE [157], FEAST [158], ALBIOS [159]), evaluated the use of albumin as a resuscitation fluid. Except for patients with traumatic brain injury, evidence suggests that albumin is well tolerated as a resuscitation fluid. However, there is no evidence to suggest that albumin offers substantial outcome benefits over crystalloid solutions, albeit that their use may result in a less positive fluid balance [160,161,162]. This was demonstrated in an RCT by Martensson et al., where resuscitation with 20% albumin decreased resuscitation fluid requirements, minimized positive early fluid balance, and was not associated with any harm compared with 4–5% albumin. The use of 5% albumin in severe burn patients requires further research [161].

Only one retrospective study involving 114 patients incorporated IAP into the respiratory and fluid management concept. This study showed that using PAL treatment (PEEP set at the level of IAP, albumin 20%, followed by Lasix^®^) was able to keep the cumulative fluid balance in check with a significant drop in IAP, EVLWI, and rise in P/F ratio. This also resulted in faster weaning from the ventilator and improved survival compared to the matched control group [87].

Wang et al., conducted an RCT in 132 patients with SAP using fresh frozen plasma as a resuscitation fluid. Fresh frozen plasma shortens the duration of positive fluid balance, decreases the overall fluid balance within 72 h, reduces the duration of mechanical ventilation and admissions to ICU, and improves PaO_2_/FiO_2_ and mortality in severe acute pancreatitis [163].

Several animal studies proved that hypertonic saline (HTS) resuscitation improves hemodynamics [164,165,166,167]. HTS treatment allows smaller fluid volume resuscitation in the burn shock period and reduces the risk of low abdominal perfusion and secondary ACS 21]. The American Burn Association evaluated the efficacy of HTS in burn patients, however, the evidence in favor is equivocal. Additional studies are required to define the correct dosage and timing [168].

### 4.3. Fluid Resuscitation Strategies

The 4 D’s of fluid therapy (drug, dosing, duration, and de-escalation) should be considered during the administration of resuscitation fluids [17,148]. Fluid requirements of critically ill patients tend to change throughout their illness, and fluid therapy should be adjusted to account for these changes. Therefore, we distinguish four phases of fluid administration (ROSE) (Figure 1): the Resuscitation phase, the Optimization phase, the Stabilization phase, and the Evacuation phase [17]. The ROSE concept may help to guide therapeutic decision-making [17].

**Figure 5:**
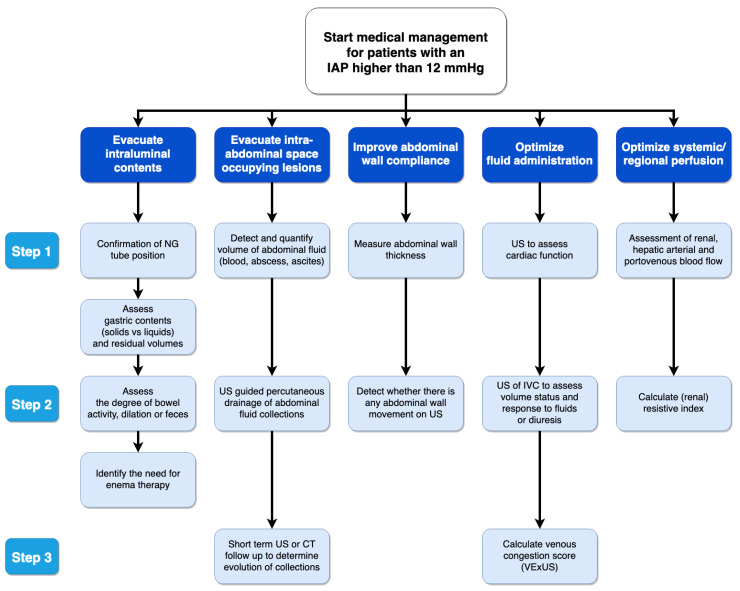
Potential use of POCUS according to WSACS medical management algorithm.

Decisions regarding the administration of intravenous fluids should be guided by functional hemodynamic measurements, such as pulse pressure or stroke volume variation. They should not be solely based on increased lactate, low MAP, or oliguria (<0.5 mL/kg/hour) [169,170]. With the increased use of ultrasound as a bedside modality in both emergency and critical care patients, it is important to consider point-of-care ultrasound (POCUS) as an adjuvant tool for IAH and management of fluid strategies (Figure 5). POCUS during the first three days of admission improved clinical performance in IAH scenarios and fluid management [171].

All of these factors should be carefully considered, to avoid the dangerous complications and vicious cycle of fluid accumulation, as illustrated in Figure 3 [172]. Fluid overload was identified as an independent risk factor for developing intra-abdominal hypertension [7,173].

The ideal rate at which fluid is administered appears to depend on how much it takes to maintain perfusion, and thus there is no clear guidance from the available literature. This would largely depend on the systemic inflammation, rate of fluid extravasation out of the intravascular compartment, and effects on cardiac function. An RCT involving 60 patients with acute pancreatitis, but without organ failure, that received either aggressive (20 mL/kg bolus followed by 3 mL/kg/h) or standard (10 mL/kg bolus followed by 1.5 mg/kg/h) resuscitation with Ringer’s lactate solution. The rate of clinical improvement was more significant with aggressive hydration, and no patients developed signs of fluid overload [156]. Another RCT in 76 patients with SAP showed that rapid, uncontrolled fluid resuscitation (10–15 mL/kg/h or until a hematocrit <35% within 48 h) significantly worsened the rates of infections, ACS, the need for mechanical ventilation, and mortality [43]. Although these studies are relatively small, they suggest an optimum therapeutic range for fluid therapy. Further research in this field is required to help determine appropriate fluid resuscitation strategies in this group, particularly whether targeting a hematocrit is helpful [43].

### 4.4. Interventions with Potential Beneficial Effects That Need Further Investigation

Fluid requirements may be reduced by ascorbic acid, which has an apparent (osmotic) diuretic effect that may lead to hypovolemia and reduced inflammatory response [174]. This was shown in a prospective, randomized study where the use of high-dose ascorbic acid led to a significantly reduced amount of resuscitation volume [175].

Peritoneal resuscitation (PR) corrected many of the physiologic derangements that lead to eventual organ dysfunction, including endothelial cell dysfunction, tissue ischemia, reduction in capillary blood flow, derangements in fluid exchange, and electrolyte handling, and increased inflammatory mediators. Studies in trauma patients have shown that PR was associated with accelerated abdominal closure, reduced abdominal complications, and reduced mortality [176]. Further research in this field is required.

### 4.5. Limitations

Although the literature search was broad, it was limited to those studies published in English. There were potential sampling errors in the search terms, and the search was limited to Scopus and PubMed. Negative studies are less likely to be published and hence would not have come to our attention during the literature search. The studies included were also heterogeneous in their sampled populations and data, making pooled analysis impossible. Future studies should broaden the search to include other languages.

Final take-home messages on the relation between fluid resuscitation and IAH:

- There is a relationship between fluid resuscitation, fluid accumulation, and secondary IAH. This signal, from the limited number of RCTs, needs further confirmation.
- Crystalloids are associated with a more positive fluid balance and a greater likelihood of developing IAH compared to colloids or hypertonic solutions.
- Fluid resuscitation in IAH may preserve cardiac output, however, it does not prevent organ damage.
- Delivery of blood products in a 3:2 ratio of RCC: FFP (red blood cells: fresh frozen plasma) and 5:1 for RCC: platelets, may reduce MOF and infectious complications, and increase ventilator-free days [63].
- Fluid resuscitation leads to IAH and venous congestion (or venous hypertension), contributing to gut edema and diminished gut contractility.
- The relationship between fluid resuscitation, fluid accumulation, and secondary IAH holds in the setting of sepsis (capillary leak), severe burn injury, emergency surgery, and trauma with the presence of the deadly triad (coagulopathy, acidosis, hypothermia).
- Fluid removal with diuretics or CVVH may restore cumulative fluid balance and may reduce IAP. The time to initiate RRT in this setting remains unclear.
- Bladder pressure measurements should be performed after infusion of more than 25 mL during the acute resuscitation phase, and one should check for peak inspiratory pressures greater than 40 cm H2O.

The presence of IAH is associated with a poor prognosis. The presence of ACS warrants escharotomy or surgical decompression of the abdominal cavity, while IAH usually responds to medical therapy [48].

## 5. Conclusions

Intravenous fluid administration plays an essential role in developing IAH and ACS. Multiple pathophysiological mechanisms have been described, notably damaging the endothelial glycocalyx. Fluid balance has been identified as an independent risk factor in several clinical studies and can contribute to the development of IAH, venous congestion, gut edema, and diminished gut contractility. Evidence identifying the best resuscitation targets and management strategies regarding type, timing, and volume of fluids in patients with IAH is scarce. It is striking how there has been little advancement of new studies or data in recent years, as the bulk of the literature is more than five years old. Therefore, further research is required to improve insights into this topic.

## Data Availability

Not applicable.

## Supplementary Materials

The following are available online at https://www.mdpi.com/article/10.3390/life12091390/s1, Table S1: Overview of included studies on burn patients, Table S2: Overview of included studies on SAP patients, Table S3: Overview of included studies on trauma patients, Table S4: Overview of included studies on medical and surgical patients.

## Abbreviations

ACS	abdominal compartment syndrome
ADH	anti-diuretic hormone
ALI	acute lung injury
APP	abdominal perfusion pressure
ARDS	acute respiratory distress syndrome
BMI	body mass index
BMT	bone marrow transplantation
BRG	burn resuscitation guidelines
CLFM	conservative late fluid management
CLI	capillary leak index
CO	cardiac output
CPB	cardiopulmonary bypass
CR	case report
CVVH	continuous veno-venous hemofiltration
CVP	central venous pressure
d	day
DL	damage control laparotomy
ECMO	extra-corporeal membrane oxygenation
ECW	extracellular body water
EGL	endothelial glycocalyx layer
eOR	emergency open repair
EVAR	endovascular aortic repair
EVLWI	extravascular lung water index
eEVR	emergency endovascular repair
FOAM	free open access medical education
FB	fluid balance
FFP	fresh frozen plasma
HES	hydroxyethyl starch
HLS	hypertonic lactated saline
IAP	intra-abdominal pressure
IAH	intra-abdominal hypertension
ICP	intra-cranial pressure
ICU	intensive care unit
ITP	intra-thoracic pressure
IV	intra-venous
L	liters
LR	ringer’s lactate solution
LOS	length of stay
M	medical
MAP	mean arterial pressure
MOF	multiple organ failure
MV	mechanical ventilation
NMB	neuromuscular blocker
NGT	nasogastric tube
NR	not reported
NS	0.9% saline
OF	organ failure
PAL	positive end-expiratory pressure, albumin, and Lasix^®^ (furosemide)
PCD	percutaneous catheter drainage
PCS	poly-compartment syndrome
PEEP	positive end-expiratory pressure
POCUS	point-of-care ultrasound
pts	patients
rAAAs	ruptured abdominal aortic aneurysms
RCC	red cell concentrate
RF	risk factor
S	surgical
SAP	severe acute pancreatitis
SAPS II	Simplified Acute Physiology Score II
SH group	combination of 0.9% saline and hydroxyethyl starch (HES)
SHG group	combination of 0.9% saline, hydroxyethyl starch and glutamine
SHINE	shock induced endotheliopathy
SN	supranormal resuscitation group
SOFA	Sequential Organ Failure Assessment Score
ST group	standard therapy
TBSA	total body surface area
TEP	trauma exsanguination protocol
TI group	targeted intervention
U	units
UO	urine output
WSACS	The Abdominal Compartment Society
