# Supplementary material for: Fluid Management, Intra-Abdominal Hypertension and the Abdominal Compartment Syndrome: A Narrative Review
Source: Life (Basel). 2022 Sep 6;12(9):1390. doi: 10.3390/life12091390 (PMC9502789; doi:10.3390/life12091390)
Supplement: Supplementary file 1 [file life-12-01390-s001.zip › life-1838627-supplementary.pdf]

Supplementary Tables

Table S1: Overview of included studies on burn

**Author** patients	**Type of study**	**LOE**	**MINOR Score**	**Mean % TBSA**	**ISS**	**Definition of IAH: WSACS or other**	**Prevalence**	**% ACS mortality**	**Correlation between IAP and IV fluid**
Ivy et al. [19]	Prospective	3	12	46 (22-80)		IAH = IAP > 25mmHg; ACS = ↓pulmonary compliance + persistent IAH + treatment of decompression	70% IAH<br>20% ACS	20	r^2=0.121
O'Mara et al. [23]	RCT	3	19	C: 50.1 ± 12.4<br>P: 52.1 ± 12.4		IAH= IAP > 25mmHg; ACS = IAP + renal or pulmonary compromise		C: 26.6<br>P: 18.75	C: r^2=0.352<br>P: r^2=0.657
Oda et al. [21]	Observational	3	18	HLS: 66.7 ± 20.1<br>LR: 64.2 ± 20.4		IAH = IAP> 22mmHg; ACS = IAH + tense abdomen + high PIP or oliguria	HLS: 14% IAH<br>LR: 50% IAH	30.5	
Oda et al. [22]	Observational	3	12	45.9 ± 21.8		IAH = IAP> 22 mmHg; ACS = IAH + tense abdomen = high PIP or oliguria	16.6% ACS		
Ennis et al. [18]	Prospective	3	19	BRG: 52 ± 17<br>control: 50 ± 17		ACS = IAH >20mmHg + MOF requiring DL	BRG: 5% ACS<br>Control: 16% ACS	BRG: 18<br>Control: 31	% TBSA ~ ACS:<br>OR, 1.052; CI, 1.009-1.097; p=0.018
Ruiz-Castilla et al. [24]	Observational	3	12	33 (25-58)		WSACS	IAH: 72%<br>ACS: 4%	38.9	
Wise et al. [26]	Observational	3	12	24.9 ± 24.9		WSACS	IAH: 78.6%<br>ACS: 28.6%	62.5	
Mbiine et al. [20]	Observational	3	13	43.2 ± 20.2		WSACS	IAH: 57.8%	82.6	RR 1.04 (0.61-1.79)
Talizin et al. [25]	Prospective	3	12	Median: 30.5		WSACS	IAH: 82.6%	63.2	r=0.29
Boehm et al. [27]	Retrospective	3	10	ACS-group:<br>50 ± 22;		WSACS		ACS: 84<br>Control: 32	

				Control group: 49 ± 21					
Hershberger et al. [28]	Retrospective	3	10	65 ± 19		WSACS		88	
Hobsen et al. [29]	Retrospective	3	10	Adult: 75<br>Pediatric:68		WSACS		Adult: 75<br>Pediatric: 50	
Markell et al. [30]	Retrospective	3	10	48 ±19	36 ± 15	ACS = IAP > 30 mmHg + urine output <30 mL/h	ACS: 26.7%	90	
McBeth et al. [31]	Retrospective	3	9	31.4 ± 20.9	21.8 ±8.3	WSACS	ACS: 80%		
Park et al. [32]	Retrospective	3	10	Pre-protocol: 39 ± 18<br>Postprotocol 38 ± 18			Pre-protocol:10%<br>Postprotocol: 3%		
Ivy et al. [33]	Case series	5		> 70		IAP > 25 mmHg + oliguria, decreased pulmonary compliance	100%	100	
Streit et al. [35]	Case report	6		80		WSACS			
Sun et al. [36]	Case report	6		70		WSACS			

MINORS: Methodological items for non-randomized studies Score; LOE: level of evidence; TBSA: total body surface area; WSACS: World Society of the Abdominal Compartment Syndrome; ACS: abdominal compartment syndrome; IV: intravenous; IAH: intra-abdominal hypertension; HES: hydroxyethyl starch; LR: lactated Ringer solution; BRG: burn resuscitation guidelines; C: crystalloid; P: plasma; ISS: injury severity score

Table S2: Overview of included studies on SAP patients

**Author**	**Type of study**	**LOE**	**MINOR Score**	**Definition of IAH: WSACS or other**	**Prevalence**	**% ACS mortality**	**Correlation between IAP and IV fluid**
Mao et al. [43]	RCT	2	20	other	Rapid fluid group: 72.2%; Controlled fluid group: 32.5%	Rapid fluid group: 30.6; Controlled fluid group: 10%	
Du et al. [41]	RCT	2	20	WSACS	HES: 0% IAH<br>LR: 33% IAH	HES: 5%<br>RL: 2%	
Ke et al. [42]	Observational	3	19	WSACS	IAH: 62%<br>ACS: 12%	16.7%	OR: 1.003 (1.001-1.006)
Zhao et al. [40]	RCT	2	20	WSACS	IAH:<br>NS group: 15%<br>SH group: 27.5%<br>SHG group: 27.5%	NS group: 12.5%<br>SH group: 5%<br>SHG group: 7.5%	
Lee et al. [45]	Case report	6					
Park et al. [46]	Case report	6					

LOE: level of evidence; SAP: severe acute pancreatitis; HES: hydroxyethyl starch; NS group: normal saline; SH group: combination of normal saline and hydroxyethyl starch (HES); SHG group: combination of normal saline, hydroxyethyl starch and glutamine

Table S3: Overview of included studies on trauma patients

Author	Type of study	LOE	MINOR Score	Population type	ISS	Definition of IAH: WSACS or other	Prevalence	% ACS mortality	Correlation between IAP and IV fluid
Raeburn et al. [51]	Observational	3	12	Trauma	ACS: 32 ± 3;<br>No ACS 27 ± 2	ACS: IAP>20 mmHg + renal or pulmonary compromise	ACS: 36%	43%	
Balogh et al. [47]	Prospective	3	18	Trauma	28 ± 3	ACS = IAH>25 mmHg + renal dysfunction	ACS: 9%	5%	
Balogh et al. [48]	Observational	3	18	Trauma	SN group: 28 ± 3;<br>N group: 27 ± 2	WSACS	SN group:<br>IAH:49.4%<br>ACS:18.8%;<br>N group:<br>IAH: 28.2%<br>ACS 11.3%	SN group: 31.2%;<br>N: group: 15.5%	
Cotton et al. [74]	Prospective	3	17	Trauma	Pre-TEP: 28 ± 15.5;<br>TEP:<br>33.3 ± 15.9	WSACS	ACS:<br>Pre-TEP: 9.9%<br>TEP: 0	Pre-TEP: 62.4;<br>TEP: 43.2	OR for TEP and MOF: 0.2 (0.106-0.395)
Neal et al. [50]	Multi-centre, Prospective	3	16	Trauma	Median 34			15.1%	C:RCC ratio > 1.5:1<br>OR: 6.2 (1.1-36)
Mahmood et al. [49]	Observational	3	21	Trauma	23 ± 10	WSACS	IAH: 74.5%<br>ACS: 0.9%	0.9%	Blood transfused<br>OR:1.11 (1.01-1.22);<br>FFP<br>OR: 1.12 (1.0-1.24);<br>Fluid:<br>OR: 1.15 (0.97-1.36)
Vatankhan et al. [52]	Observational	3	12	Trauma		WSACS	ACS: 28%	75%	
Gracias et al. [54]	Retrospective	3	9	Trauma		WSACS		60	

Balogh et al. [53]	Retrospective	3	10	Trauma	SN:28 ± 3;<br>N: 27 ± 2	WSACS	SN:<br>IAH: 49%<br>ACS: 18.8%<br>N:<br>IAH: 28%<br>ACS: 11%		
He et al. [55]	Retrospective	3	10	Trauma	23.1 ± 7.4		IAH: 9.7%<br>ACS: 1.1%	40	
Hwabejire et al. [56]	Retrospective	3	12	Trauma	ACS: 37 ± 14<br>No-ACS: 32 ± 14	WSACS	ACS: 6.2%	37.7	OR: 1.003 (1.002-1.004)
Joseph et al. [57]	Retrospective	3	12	Trauma		WSACS	ACS: 2.2%	55.6%	OR:1.07 (1.01-1.1)
Macedo et al. [58]	Retrospective	3	10	Trauma	21.9 ± 14.3	WSACS		60%	
Shaheen et al. [62]	Retrospective	3	10	Trauma	22.5 ± 15.3	WSACS		32.14%.	
Madigan et al. [59]	Retrospective	3	12	Trauma	ACS:<br>25.6 ± 9.06<br>control:<br>21.4 ±<br>11.02	IAH >25mmHg + organ failure		60%	Prehospital fluid:<br>OR: 1.99 (1.07-3.73)<br>ED crystalloid<br>OR: 1.85 (1.08-3.15)
Maxwell et al. [60]	Retrospective	3		Trauma	ISS 25 ± 3		4% ACS		
Rodas et al. [61]	Retrospective	3	9	Trauma		WSACS	ACS: 0.13%	0%	
Strang et al. [75]	Retrospective	3	12	Trauma	21 (13-34)	WSACS	IAH: 10.2%<br>ACS: 51.7%	25.9%	OR: 1.17-1.21
Zaydfudim et al. [69]	Retrospective	3	12	Trauma	Pre-TEP:<br>28 ± 15;<br>TEP:<br>41 ± 18		Pre-TEP: 20%<br>TEP: 0%		

Kopelman et al. [65]	Case series	5		Trauma				66.7%	
Kula et al. [72]	Case series	5		Trauma					
Chamisa et al. [64]	Case report	6		Trauma					
Michel et al. [66]	Case report	6		Trauma					
Kobayashi et al. [71]	Case report	6		Trauma					
Perks et al. [68]	Case report	6		Trauma					
Morell et al. [67]	Case report	6		Trauma					
Burrows et al. [63]	Case series	5		Trauma/ Surgery					
Parra et al. [34]	Case report	6		Burn/ Trauma					
DeCou et al. [70]	Case series	5		Trauma/ sepsis					
Jensen et al. [37]	Case series	5		Burn/ Trauma					

Table S4: Overview of included studies on medical and surgical patients

Author	Type of study	LOE	MINOR Score	Population type	Definition of IAH: WSACS or other	Prevalence	% ACS mortality	Correlation between IAP and IV fluid
Divarci et al. [81]	Prospective	3	12	Medical	WSACS	IAH: 9%<br>ACS: 4%	16%	
Ranjit et al. [84]	Prospective	3	16	Medical	NR	ST-group: 30%<br>TI-group: 7.9%		
Daugherty et al. [86]	Observational	3	12	Medical	WSACS	IAH: 85%<br>ACS: 25%	80%	
Cordemans et al. [78]	Observational	3	11	Medical	WSACS	IAH: 20%		
Dorigatti et al. [85]	Observational	3	12	Medical	WSACS	IAH: 68%<br>ACS: 28%	71.42%	
Cordemans et al. [87]	Retrospective	3	10	Medical	WSACS			
Pupelis et al. [44]	Retrospecitve	3	10	Medical	WSACS	IAH: 68.5%	12.4%	
Struck et al. [79]	Retrospective	3	10	Medical	WSACS	ACS: 17.2%	100%	
Kula et al. [73]	Case series	5		Medical				
Macalino et al. [77]	Case report	6		Medical				
Tsuang et al. [76]	Case report	6		Medical				
Dauplaise et al. [80]	Case report	6		Medical				
Gala et al. [82]	Case report	6		Medical				
Biancofiore et al. [92]	Observational	3	13	Surgical		IAH: 32%	14.7%	
Šerpytis et al. [97]	Observational	3	12	Surgical	WSACS	IAH: 45.5%		POD1: r=0.492<br>POD2: r=0.518<br>POD3: r=0.405
Makar et al. [95]	Prospective	3	18	Surgical		ACS: 6.7%	6.7%	r=0.43
Dalfino et al. [93]	Observational	3	14	Surgical	WSACS	IAH: 31.8%		OR 4.31 (1.68-5.54)
Muturi et al. [96]	Observational	3	13	Surgical	WSACS	IAH: 60.5%<br>ACS: 20%		OR 1 (1.0-1.002)
Kotlińska-Hasiec et al. [94]	Observational	3	20	Surgical	NR			r=0.57

McNelis et al. [99]	Retrospective	3	10	Surgical	NR		66.7%	$P=1/(1+e^{-z})$
Rubenstein et al. [89]	Retrospective	3	10	Surgical	NR	ACS: 29%	66.7%	
Leclerc et al. [98]	Retrospective	3	10	Surgical	WSACS	ACS :17%	37.5%	
Miranda et al. [88]	Retrospective	3	10	Surgical	WSACS	ACS: 12%	67%	
Fietsam et al. [101]	Case report	6		Surgery				
De Wolf et al. [100]	Case report	6		Surgery				
Bressan et al. [91]	Case report	6		Surgery				
Rabbi et al. [102]	Case report	6		Surgery				
Shiiya et al. [103]	Case report	6		Surgery				
Biffl et al. [104]	Prospective	3	10	Medical-surgical	WSACS		38% in trauma;<br>100% in non-trauma	
Malbrain et al. [110]	Observational	3	12	Medical-surgical	WSACS	IAH: 58.8%<br>ACS: 8.2%		
Malbrain et al. [109]	Observational	3	14	Medical-surgical	WSACS	IAH: 32.1%<br>ACS: 4.2%	27.5%	
Dalfino et al. [107]	Observational	3	14	Medical-surgical	WSACS	IAH: 30.1%	54%	OR:5.22 (2.03-7.45)
Vidal et al. [113]	Prospective	3	14	Medical-surgical	WSACS	IAH 64%<br>ACS 6%	100%	RR 2.5 (0.91-6.9)
Blaser et al. [116]	Observational	3	14	Medical-surgical	WSACS	IAH 32.3%<br>ACS 6%		
Kim et al. [114]	Observational	3	14	Medical-surgical	WSACS	IAH 42%<br>ACS 2%	0%	r^2:0.48
Iyer et al. [111]	Observational	3	13	Medical-surgical	WSACS	IAH: 39%<br>ACS: 2%		OR: 2.678 (1.48-4.84)
Malbrain et al. [115]	Systematic review	1		Medical-surgical				
Dąbrowski et al. [5]	Observational	3	11	Medical-surgical	WSACS	IAH: 28%		
Murphy et al. [108]	Observational	3	14	Medical-surgical	WSACS	IAH: 45%<br>ACS 2.8%	87.5%	OR 2.45 (1.18-5.14)
Blaser et al. [112]	Observational	3	14	Medical-surgical	WSACS	IAH: 48.9%<br>ACS : 6.3%	67.7%	OR: 1.168 (1.068-1.278)
Aik-Yong et al. [105]	Retrospecitve	3	10	Medical-surgical	WSACS	ACS: 0.1%	47.1%	

Cothren et al. [106]	Retrospective	3	10	Medical-surgical	WSACS		38.9%	
Britt et al. [38]	Retrospecitve	3	9	Trauma-burn	NR		60%	
Reed et al. [39]	Retrospective	3	10	Trauma-burn	NR			

ED: emergency department, ST group: standard therapy; TI group: targeted intervention; z= -18,6763 + 0,1671 (peak airway pressure) + 0,0009 (fluid balance)
